# Population genetic structure of *Aedes aegypti* subspecies in selected geographical locations in Sudan

**DOI:** 10.1038/s41598-024-52591-6

**Published:** 2024-02-05

**Authors:** Sara A. Abuelmaali, Abadi M. Mashlawi, Intan Haslina Ishak, Mustafa Fadzil Farid Wajidi, Zairi Jaal, Silas Wintuma Avicor, Nur Faeza Abu Kassim

**Affiliations:** 1 129 Medical Entomology Laboratory, School of Biological Sciences, Universiti Sains Malaysia, 11800 Penang, Malaysia; 2 National Public Health Laboratory, Federal Ministry of Health, Khartoum, 11115 Sudan; 3 Department of Biology, College of Science, Jazan University, P.O. Box. 114, Jazan, 45142 Kingdom of Saudi Arabia; 4 Molecular Entomology Research Group, Universiti Sains Malaysia, 11800 Penang, Malaysia; 5 Vector Control Research Unit, School of Biological Sciences, Universiti Sains Malaysia, 11800 Penang, Malaysia; 6 Entomology Division, Cocoa Research Institute of Ghana, New Tafo-Akim, Ghana

**Keywords:** Ecology, Evolution, Genetics, Molecular biology

## Abstract

Although knowledge of the composition and genetic diversity of disease vectors is important for their management, this is limiting in many instances. In this study, the population structure and phylogenetic relationship of the two *Aedes aegypti* subspecies namely *Aedes aegypti aegypti* (*Aaa*) and *Aedes aegypti formosus* (*Aaf*) in eight geographical areas in Sudan were analyzed using seven microsatellite markers. Hardy–Weinberg Equilibrium (HWE) for the two subspecies revealed that *Aaa* deviated from HWE among the seven microsatellite loci, while *Aaf* exhibited departure in five loci and no departure in two loci (A10 and M201). The Factorial Correspondence Analysis (FCA) plots revealed that the *Aaa* populations from Port Sudan, Tokar, and Kassala clustered together (which is consistent with the unrooted phylogenetic tree), *Aaf* from Fasher and Nyala populations clustered together, and Gezira, Kadugli, and Junaynah populations also clustered together. The Bayesian cluster analysis structured the populations into two groups suggesting two genetically distinct groups (subspecies). Isolation by distance test revealed a moderate to strong significant correlation between geographical distance and genetic variations (*p* = 0.003, *r* = 0.391). The migration network created using divMigrate demonstrated that migration and gene exchange between subspecies populations appear to occur based on their geographical proximity. The genetic structure of the *Ae. aegypti* subspecies population and the gene flow among them, which may be interpreted as the mosquito vector's capacity for dispersal, were revealed in this study. These findings will help in the improvement of dengue epidemiology research including information on the identity of the target vector/subspecies and the arboviruses vector surveillance program.

## Introduction

The rapid shift of arboviral diseases burden is concerning, particularly in Africa, where most people are impoverished, and health services are preoccupied with the malaria burden. However, in Sudan, in addition to substantiating the shift from the high burden of malaria parasites to arboviral diseases, multiple reports from various parts of the country indicate a massive increase in arboviral disease cases and expansion according to the Federal Ministry of Health reports^1,2^.

Dengue, yellow fever, chikungunya and other arboviral diseases are endemic in Sudan^2^. These arboviruses are transmitted to humans via the bites of infected mosquitoes of the genus *Aedes*, subgenus *Stegomyia*, particularly *Aedes aegypti*, which plays a major role in transmitting arboviral diseases. In the country, entomological surveillance revealed that, *Ae. aegypti* is the predominant mosquito in all arbovirus’ endemic areas in the country^3–6^.

Wide ecological research has been conducted on the population structure and dynamics of insects and this can indicate future population trends. Predicting outbreaks requires an understanding of the relationship between population structure and change in response to anticipated environment changes. Hence, population genetics research may reveal important details about a species' dispersion and population dynamics^7^.

Microsatellites are one of the most potent tools produced in recent years in population structure and population genetics, among many molecular markers available^8^. Microsatellites are highly variable genetic markers that have frequently been used in population genetic investigations at the intraspecific level because of their high polymorphism, co-dominant inheritance ease and reliability of scoring alleles, high abundance, and highly changeable nature of their loci in the genome. Therefore, microsatellites are widely used as common markers in insect studies^9–11^.

Several studies on the genetic structure of *Ae. aegypti* have been conducted using microsatellite markers in different parts of the world such as the Pacific region^12^, China^13^, United States of America^14^, Philippines^15^, Sri Lanka^16^, Black Sea^17^, Kenya^18^, Sudan^3^ and several others. A global study reviewed the genetic variation at 12 microsatellite loci in 79 *Ae. aegypti* populations from 30 countries across six continents to infer historical and modern invasion patterns. Their findings verified the genetic departure of the two subspecies *Ae. aegypti formosus* and *Ae. aegypti aegypti*^19^.

The control of mosquito-borne diseases has primarily been accomplished by vector control, most typically by killing the vectors with various biocides. However, control programs based on this technique have been universally considered ineffective due to the rise in resistance^20,21^. Estimating the genetic composition of the *Ae. aegypti* population and gene flow would help researchers better understand dengue epidemiology. Molecular genotyping of the mosquito vector using these markers has revealed new information on the vectors especially microevolution, exposing the populations’ gene flow pattern, which may be interpreted as the vector's ability to disperse^22,23^.

Eastern Sudan witnessed the greatest chikungunya epidemic in Africa to date in 2018/19, affecting roughly 500,000 people, with the *Aedes aegypti* vector being the most prominent vector in the outbreak areas^24^. The Sudanese Ministry of Health recorded 3326 cases of dengue fever in 8 Sudanese States on November 23, 2022 with the disease claiming the lives of 23 individuals. The largest dengue fever outbreak to hit Sudan in almost a decade is currently occurring, with Red Sea State and North and South Kordofan being particularly heavily struck^25^.

*Aedes aegypti*, which is thought to have originated in Africa, is known to have two subspecies or variants that differ in terms of behaviour, transmitting capacity, and distribution^26^^,^^27^. In Sudan, both *Aedes aegypti* subspecies are common and have a wide range of distribution. However, the *Aedes aegypti aegypti* (*Aaa*) subspecies seems to be more prevalent in the east of the country while the *Aedes aegypti formosus* (*Aaf)* subspecies is the most common form of the vector in western Sudan^1,3^. Only a few studies have reported the subspecies genetic differences in Sudan, and they indicated that the genetic structure of the two subspecies were clearly different from one another^1,3,28^.

The study of disease vectors' genetic structure and variability sheds crucial light on their biology, behaviour, genetic assimilation, and ability to transfer diseases. A previous study on the distinct populations of *Aedes aegypti* subspecies in different areas of Sudan using CO1 mitochondrial marker observed that the two subspecies were phylogenetically structured into two clusters^1^. However, another study using the ND4 mitochondrial gene, indicated gene flow among the populations of the *Aedes aegypti* subspecies, suggesting that they are not entirely genetically isolated^28^.

Hence, understanding the genetic characteristics of the two forms of *Aedes aegypti* is required for a better understanding of the biology, behaviour, genetic mixing, and disease-transmission potential of the vectors. In this research, *Aedes aegypti* subspecies populations were sampled from eight sites in Sudan to examine the genetic structure and diversity of the populations using seven microsatellite markers.

## Results

### Mosquito identification and genetic variability

**Figure 1:**
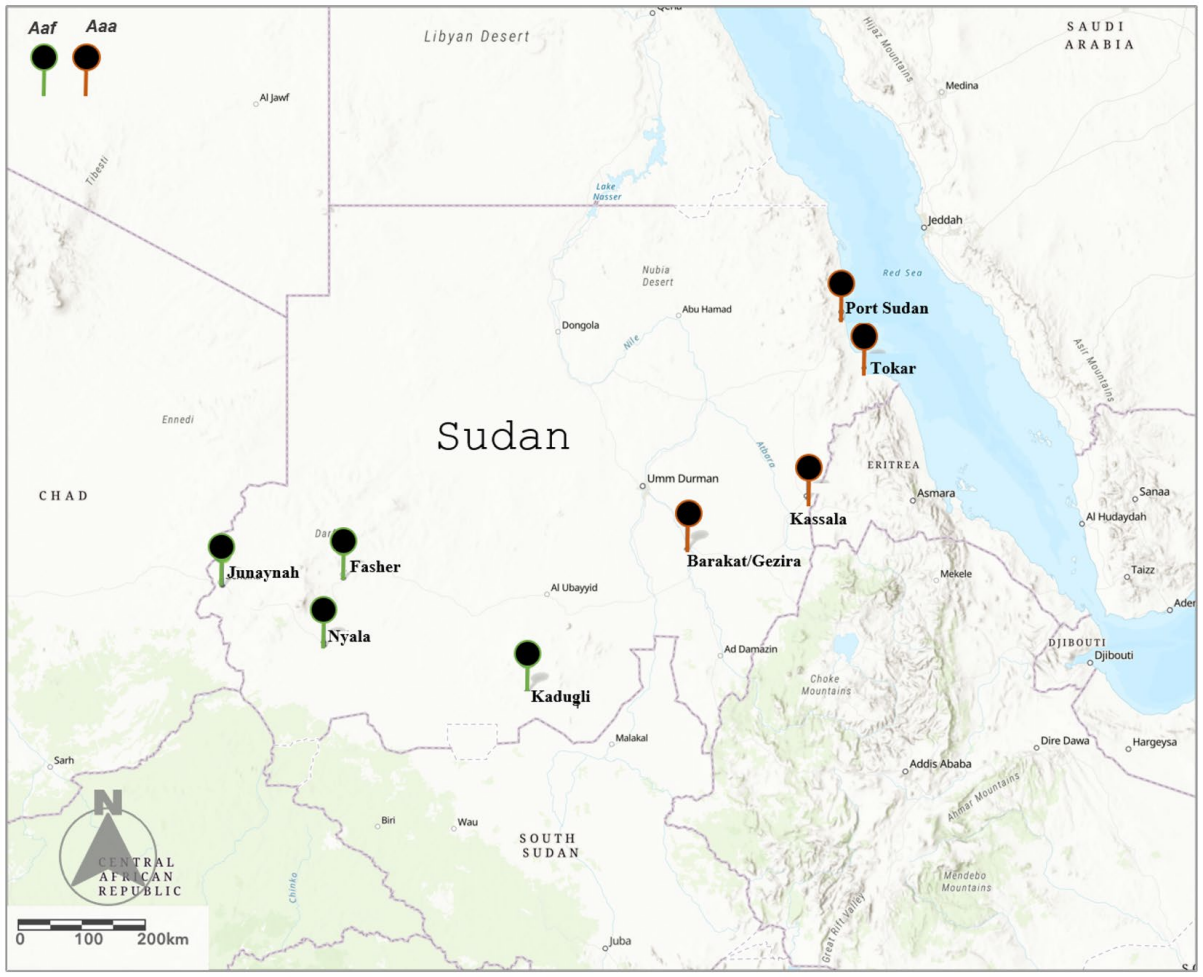
Map showing the eight study sites of *Aedes aegypti* subspecies in Sudan. *Aaa, Aedes aegypti aegypti. Aaf, Aedes aegypti Formosus*.

The results of the identification showed that the *Aedes aegypti* mosquitoes from the western and southern parts of the county (Darfur and Kordofan) thus Nyala (N), Al Fasher (F), Al Junaynah (J), and Kadugli (D) were *Aaf*. Samples from each of the four towns located in the eastern and central parts of the county, namely Port Sudan (P), Tokar (T), Kassala (K), and Barakat/Gezira (G), were morphologically identified as the *Aaa* subspecies (Fig. 1).

At the seven different microsatellite loci, 202 *Aedes aegypti* mosquitoes from eight different sites were genotyped. Not all the loci were successfully amplified in all the examined locations, and the number of genotyped individuals per loci varied from 5 to 31 (Table 1). There was no evidence of scoring errors due to significant allele dropout or stuttering as well as no proof of null alleles presence in all loci. All the seven loci were polymorphic, albeit at varying degrees, with the number of alleles per locus ranging from 14 at locus A10 (AR = 3.4) to 37 at locus B19 (AR = 3.8) (Table 2). The average number of alleles across the seven loci in the populations ranged from 8.7 ± 4.27 in Fasher to 14.3 ± 6.18 in Kassala, with an average of 12.3 ± 2.16 alleles per locus (Table 1).

**Table 1:** Number of alleles means and total number in eight populations within seven loci.

Locus	P	T	K	G	D	N	F	J	Mean	SD	Total
A10	7	10	9	12	11	11	10	9	9.88	1.55	14
B07	21	16	24	25	20	23	13	21	20.38	4.07	35
H08	9	7	10	13	14	12	7	9	10.13	2.64	19
G11	6	7	10	9	15	10	3	14	9.25	3.99	41
B19	19	12	22	18	18	21	15	17	17.75	3.20	37
M313	12	6	14	10	9	10	5	9	9.38	2.93	19
M201	7	9	11	10	9	12	8	9	9.38	1.60	18
Mean	11.57	9.57	14.29	13.86	13.71	14.14	8.71	12.57	12.30	2.16	26.14
SD	6.11	3.51	6.18	5.76	4.31	5.46	4.27	4.89	5.06	0.97	11.05

P: PortSudan; Tokar: K: Kassala, G: Gezira, D: Kadugli, N: Nyala, F: Fasher, J: Junainah.

**Table 2:** Summary statistics of number of individuals genotyped (N), allelic range and richness (*A*_R_), number of alleles (*N*_A_) and genetic diversity at each locus and each population of *Ae. aegypti.*

Study site	Locus	A10	B07	H08	G11	B19	M313	M201	Total/average
Port Sudan	N	15	21	21	12	21	14	20	124/17.7
	N_A_	7	21	9	6	19	12 (1)	7	81/11.57
	Allelic range	8	26	15	38	29	14	8	–
	A_R_	2.938	3.782	2.794	2.966	3.585	3.408	3.200	2.834
	Gene diversity	0.783	0.962	0.743	0.799	0.924	0.887	0.846	0.849
Tokar	N	23	29	24	19	19	15	17	132/18.86
	N_A_	10	16 (1)	7	7	12 (1)	6	9	67/9.57
	Allelic range	9	24	9	40	29	8	11	–
	A_R_	3.215	3.545	2.839	2.720	3.314	2.775	3.108	3.07
	Gene diversity	0.848	0.919	0.762	0.717	0.871	0.748	0.822	0.812
Kassala	N	18	29	27	12	18	16	19	139/19.86
	N_A_	9	24	10 (1)	10 (1)	22	14 (1)	11	99/14.14
	Allelic range	13	32	9	38	30	16	10	–
	A_R_	3.144	3.764	3.043	3.062	3.776	3.568	3.395	3.39
	Gene diversity	0.830	0.961	0.812	0.811	0.963	0.923	0.887	0.883
Gezira	N	23	24	22	5	18	18	16	126/18
	N_A_	12	25	13 (2)	9 (3)	18 (3)	10	10	96/13.7
	Allelic range	11	30	18	35	33	14	12	–
	A_R_	3.405	3.794	3.471	3.867	3.635	3.166	3.387	3.531
	Gene diversity	0.891	0.966	0.905	0.975	0.941	0.837	0.890	0.915
Kadugli	N	22	21	19	13	19	16	9	119/17
	N_A_	11	20	14	15 (8)	18 (1)	9	9 (2)	96/13.7
	Allelic range	10	25	18	63	30	12	16	–
	A_R_	3.514	3.737	3.309	3.603	3.682	3.006	3.041	3.413
	Gene diversity	0.912	0.954	0.865	0.929	0.944	0.790	0.792	0.883
Nyala	N	18	31	17	6	21	24	20	137/19.57
	N_A_	11	23(1)	12 (2)	10 (6)	21(1)	10 (1)	12 (1)	99/14.14
	Allelic range	13	34	24	48	28	15	14	–
	A_R_	3.425	3.734	3.460	3.818	3.788	2.958	3.387	3.15
	Gene diversity	0.895	0.955	0.901	0.983	0.963	0.785	0.887	0.91

*N* number of individuals genotyped, *NA* number of alleles observed in each sample with number of private alleles in parenthesis, *Allelic range* number of repeat units that alleles span, *AR* allelic richness and *Gd* gene diversity.

Private alleles (restricted to a single population) were observed at all loci except A10 locus and accounted for 46 of the 183 alleles (25.1%) recorded across all loci at all sites, while G11 recorded the highest number of private alleles. The greatest number of private alleles was observed at Kadugli and Nyala with 7 private alleles in both, followed by Junaynah and Gezira with 8 private alleles (Table 2). All microsatellite loci of the *Ae. aegypti* populations were found to be polymorphic with the average number of alleles per locus ranging from 9.25 (G11) to 20.38 (B07) (Table 2).

**Figure 2:**
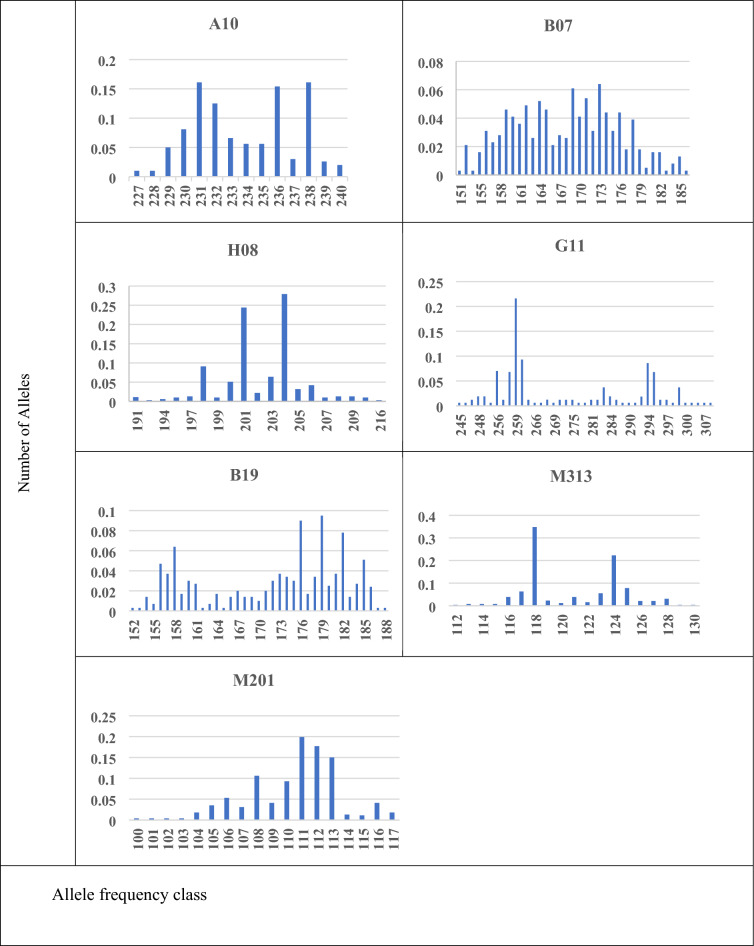
Allele frequency distribution for eight populations of *Ae. aegypti* across seven microsatellite loci.

The number of alleles (NA), allelic range, allelic richness (AR), and gene diversity (Gd) were used to evaluate genetic diversity, and the results showed variations across loci and sites (Table 2). Although allelic richness showed variation among different sites and loci, the average AR seemed to be consistent, ranging from 3.08 in M313 to 3.79 in B07. Generally, all the sites showed a relatively high gene diversity, ranging from 0.812 to 0.915. Barakat/Gezira had the highest average gene diversity (0.949) between sites (Fig. 2).

### Hardy–Weinberg equilibrium (HWE), linkage disequilibrium (LD), and F_IS_ among the eight populations of *Aedes aegypti*

All loci showed significant deviations from HWE equilibrium (in one population or more) except M201 locus which followed HWE. Generally, 14 out of 56 tests (25%) significantly departed from equilibrium after Benjamini–Hochberg multiple testing correction (Table 3). Port Sudan, Kadugli and Junaynah populations showed non-significant deviation from HWE, which probably means that those populations were not following HWE (Table 3). Significant deviation from linkage disequilibrium was found in 39 of the 168 pairwise comparisons between individual loci at each site (23.2% of tests performed) (Table 4).

**Table 3:** Summary statistics of microsatellite data of eight populations of *Ae. aegypti* from Sudan.

Study site	Locus	A10	B07	H08	G11	B19	M313	M201	Average
Port Sudan	H_O_	1.000	1.000	0.905	0.750	1.000	1.000	1.000	0.951
	H_E_	0.791	0.963	0.747	0.797	0.926	0.892	0.852	0.853
	HWE p value	1.000	1.000	1.000	1.000	0.077	0.497	0.14	–
	F_IS_	− 0.277	− 0.040	− 0.219	0.062	− 0.083	− 0.127	− 0.182	− 0.124
Tokar	H_O_	1.000	0.966	1.000	1.000	0.895	0.800	1.000	0.952
	H_E_	0.851	0.920	0.767	0.725	0.872	0.749	0.827	0.816
	HWE p value	1.000	0.021*	0.35	0.028*	0.133	0.441	0.588	–
	F_IS_	− 0.181	− 0.051	− 0.313	− 0.394	− 0.027	− 0.070	− 0.217	0.814
Kassala	H_O_	1.000	0.862	0.963	0.833	0.900	0.938	1.000	0.982
	H_E_	0.835	0.960	0.815	0.812	0.962	0.923	0.891	0.885
	HWE p value	0.329	0.035*	0.007*	0.014*	1.000	1.000	1.000	–
	F_IS_	− 0.205	− 0.103	− 0.187	− 0.028	0.066	− 0.016	− 0.127	− 0.086
Gezira	H_O_	0.913	0.875	0.864	1.000	0.722	0.899	0.875	0.878
	H_E_	0.892	0.965	0.904	0.978	0.935	0.838	0.889	0.914
	HWE p value	0.014*	1.000	0.014*	1.000	0.014*	0.035*	0.714	
	F_IS_	− 0.025	0.095	0.046	− 0.026	0.233	− 0.063	0.017	0.041
Kadugli	H_O_	1.000	1.000	1.000	0.923	0.947	1.000	0.889	0.966
	H_E_	0.914	0.955	0.869	0.929	0.945	0.796	0.797	0.886
	HWE p value	1.000	1.000	0.126	1.000	1.000	0.077	1.000	
	F_IS_	− 0.096	− 0.049	− 0.155	0.007	− 0.003	− 0.265	− 0.123	0.098
Nyala	H_O_	0.889	0.903	1.000	0.833	1.000	0.958	0.950	0.933
	H_E_	0.895	0.954	0.904	0.980	0.964	0.789	0.889	0.911
	HWE p value	1.000	0.028*	0.077	1.000	0.861	0.014*	1.000	
	F_IS_	0.007	0.054	− 0.110	0.153	− 0.038	− 0.220	− 0.071	0.032
Fasher	H_O_	0.941	0.947	1.000	1.000	0.923	1.000	1.000	0.973
	H_E_	0.872	0.915	0.765	0.833	0.945	0.649	0.869	0.835
	HWE p value	0.118	0.049*	0.000*	1.000	0.525	0.004*	0.393	
	F_IS_	− 0.08	− 0.037	− 0.327	− 0.333	0.024	− 0.583	− 0.161	0.214
Junaynah	H_O_	1.000	1.000	1.000	0.923	0.941	0.929	1.000	0.97
	H_E_	0.843	0.962	0.857	0.945	0.948	0.762	0.863	0.883
	HWE p value	0.720	0.442	0.189	0.151	0.128	0.116	0.503	−
	F_IS_	− 0.194	− 0.041	− 0.174	0.024	0.008	− 0.229	− 0.171	0.111

N, number of individuals genotyped; HO, observed heterozygosity; HE, expected heterozygosity; HWE p value, test for deviation from Hardy–Weinberg Equilibrium; FIS, inbreeding coefficient; W/T p value, significance of Wilcoxon’s 2-tailed test for deviation from population mutation-drift equilibrium; bold indicates P < 0.05, bold * indicates significance after Benjamini–Hochberg multiple testing correction at α = 0.05.

**Table 4:** Linkage disequilibrium between pairs of microsatellite loci of *Ae. aegypti* populations*.*

Locus	A10	B07	H08	G11	B19	M313	M201
Port Sudan	1	0	1	0	0	1	1
Tokar	5	3	2	2	5	4	3
Kassala	1	2	1	1	2	3	2
Gezira	0	1	2	2	2	3	2
Kadugli	0	0	2	3	1	2	2
Nyala	0	0	0	1	2	1	2
Fasher	1	0	0	1	3	3	2
Junaynah	0	0	0	0	1	1	0

The inbreeding coefficient (F_IS_) over all loci demonstrated that the majority of populations revealed an excess of observed heterozygotes (many negative values). A high inbreeding rate was observed within these populations (F_*IS*_ average ranged from 0.021 to 0.179) (Table 3).

### Genetic diversity, LD, HWE and F_IS_ for the two subspecies of *Ae. aegypti*

In the *Aaa* subspecies, the p-value was significant across the 7 loci indicating deviation from HWE, while linkage disequilibrium was identified in 10 out of 21 pairs (47.6%), with inbreeding factor (F_IS_ average) = − 0.077 and moderate to low F_*ST*_ value (0.023). In the *Aaf* subspecies, HWE demonstrated departure in all loci except A10 and M201, with linkage disequilibrium noted in 7 out of 21 pairs (33%), F_*ST*_ = 0.019 and higher average inbreeding (-0.086) (Table 5). The Wilcoxon sign-rank test and mode shift test revealed no possibilities of recent population bottleneck in all the populations. All loci fit T.P.M., mutation-drift equilibrium, normal L-shaped distribution since the probability (one tail for H excess) is around 1 in all populations (non-significant p-value > 0.05) (Table 3).

**Table 5:** Summary statistics of microsatellite data in the two subspecies of *Ae. aegypti* from Sudan.

Subspecies	Locus	A10	B07	H08	G11	B19	M313	M201
Domestic form (*Aaa*)	N	38	86	39	32	71	42	37
	AR mean	3.309						
	H_O_	0.975	0.922	0.936	0.894	0.885	0.905	0.970
	H_E_	0.871	0.958	0.817	0.815	0.933	0.862	0.872
	HWE p value	0.000	0.000	0.000	0.000	0.000	0.000	0.000
	F_*IS*_ mean	− 0.077						
	F_*ST*_ mean	0.023						
Wild form (*Aaf*)	N	41	77	42	42	71	33	38
	AR	3.358						
	H_O_	0.959	0.956	1.000	0.912	0.957	0.969	0.958
	H_E_	0.889	0.962	0.857	0.958	0.960	0.757	0.887
	HWE p value	0.441	0.002	0.000	0.002	0.001	0.000	0.386
	F_*IS*_ mean	− 0.086						
	F_*ST*_ mean	0.019						

N, number of individuals genotyped; HO, observed heterozygosity; HE, expected heterozygosity; HWE p value, test for deviation from Hardy–Weinberg Equilibrium; FIS, inbreeding coefficient; bold * indicates significance after Benjamini–Hochberg multiple testing correction at α = 0.05.

### Molecular variation and differentiation in *Ae. aegypti* populations

Hierarchical AMOVA was initially performed on the two groups of *Aedes aegypti*: *Ae. aegypti aegypti* were from Port Sudan, Tokar, Kassala and Gezira populations while *Ae. aegypti formosus* were from Kadugli, Nyala, Fasher and Junaynah populations. The variance components in this comparison revealed a high percentage within populations (96.02%) compared with variation among groups (2.23%) (Table 6). Both F_CT_ (diversity between groups) estimate (F_CT_ = 0.0224), and F_SC_ (diversity among populations within a group) value (F_SC_ = 0.018) were significant (p < 0.05).

**Table 6:** Hierarchical analysis of molecular variance (AMOVA) of the allele frequencies of seven microsatellite loci in two subspecies (groups) of eight *Ae. aegypti* populations.

Grouping of populations	Source of variation	Sum of squares	Variance components	Percentage variation
Two groups according to morphological identification Group 1: P, T, K and G Group 2: D, N, F, and J	Among groups	12.45	0.071	2.24
	Among populations Within groups	28.75	0.064	1.74
	Within populations	828.68	3.05	96.02
	Total	869.884	3.18	
F statistics	F_*ST*_: 0.040* F_*SC*_: 0.018* F_*CT*_: 0.023*			

*Significant (p<0.05). P (Port Sudan), T (Tokar), K (Kassala), G (Gezira), D (Kadugli), N (Nyala), F (Fasher), J (Junaynah).

**Figure 3:**
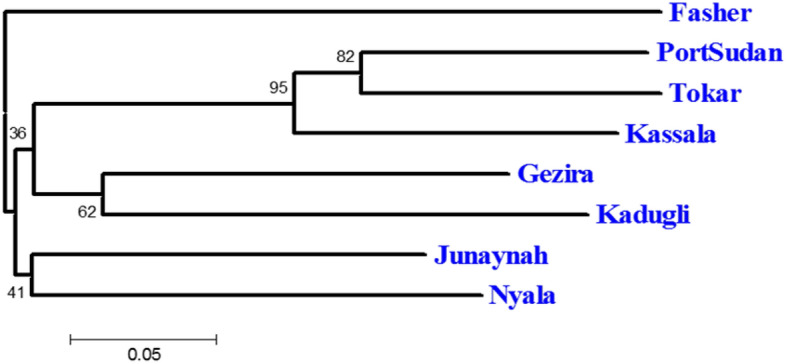
Unrooted neighbour-joining tree based on DA genetic distance at seven nuclear microsatellites of *Ae. aegypti* from eight sites in Sudan, numbers at the nodes are percentage bootstrap support from 1000 replicates. The scale bar represents 5% sequence divergence.

The isolation by distance test between all population pairs (Mantel test) was highly significant (p = 0.003) with a moderate relationship (correlation coefficient r = 0.391). Thus the correlation between geographical and genetic distance matrices advocated that landscape features may have some influence on the genetic differentiation (Fig. 3).

**Figure 4:**
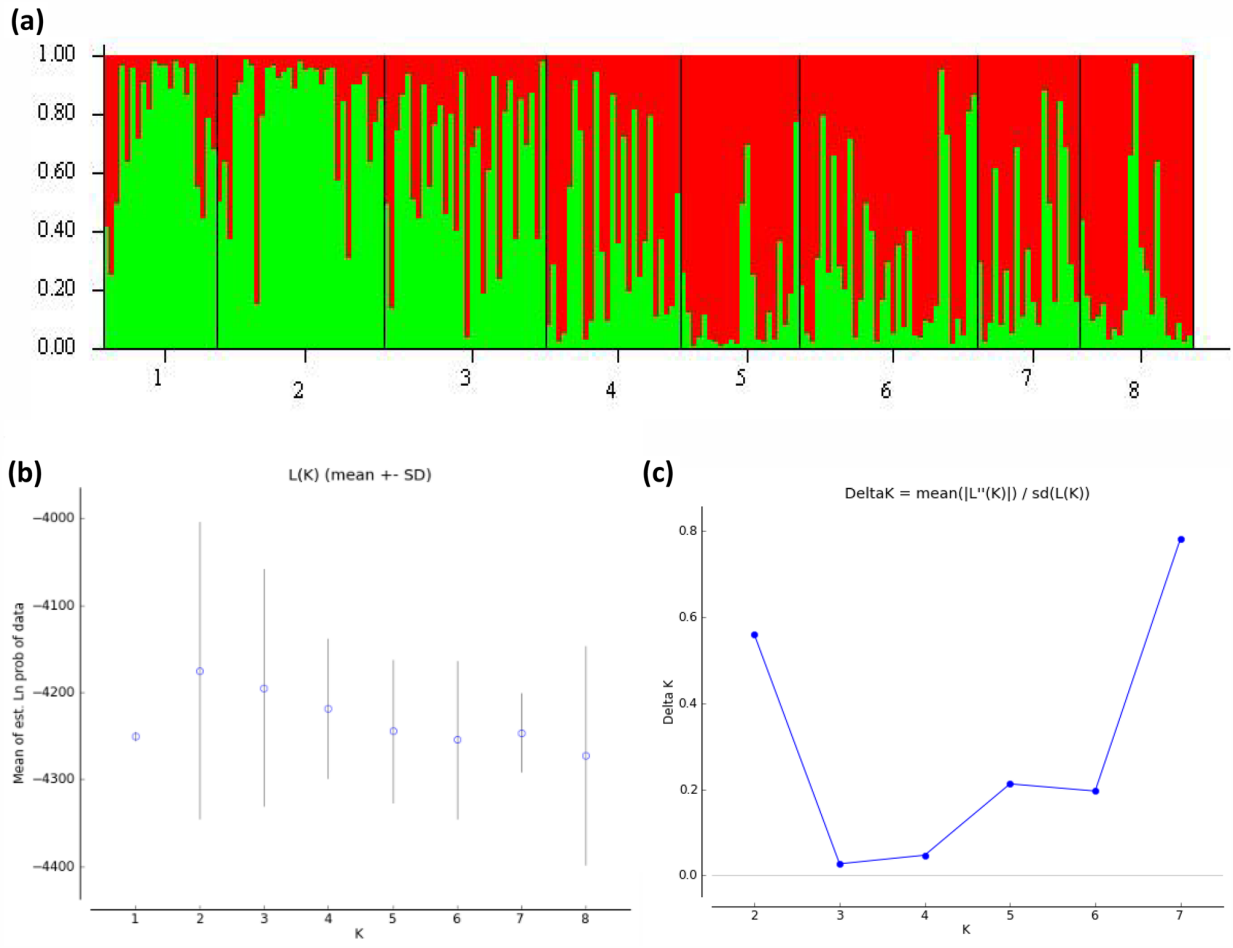
Bayesian clustering analysis generated through STRUCTURE and STRUCTURE HARVESTER based on eight microsatellite loci of eight *Ae. aegypti* populations (reduce analysis of ‘pure’ Group 1) to determine the exact value of K. (**a**) Results of assignment tests for numbers of clusters K = 2 indicated along the x-axis. (**b**) Mean (± SD) log posterior probabilities (**c**) estimate of ΔK for each value of K (putative number of populations. Each vertical line represents one individual, and y coordinates denote each individual's percentage assignment to each of the genetic clusters, represented by a different colour. Numbers from 1–8 are the study sites, 1 Port Sudan, 2 Tokar, 3 Kassala, 4 Barakat/Gezira, 5 Kadugli, 6 Nyala, 7 Fasher and 8 Junaynah.

The Migration network using divMigrate and based on Nm estimates revealed strong gene flow between Port Sudan, Kassala and Tokar *Aaa* populations which are geographically located in eastern Sudan, as well as between Nyala and Fasher populations (*Aaf* populations). The relative migration values were found to be the most between Tokar and Kassala populations and Fasher and Nyala populations (Fig. 4). STRUCTURE analysis was then performed and according to Wright's values, the overall F_*ST*_ = 0.03981 revealed moderate genetic differentiation. Generally, F_*ST*_ values among *Aedes aegypti* samples across the eight study sites were low (0.00–0.045) (Table 7).

**Table 7:** Population pairwise F_*ST*_ of the allele frequencies of seven microsatellite loci in two subspecies (groups) of eight populations of *Aedes aegypti* from Sudan.

Site (form)	P (*Aaa*)	T (*Aaa*)	K (*Aaa*)	G (*Aaa*)	D (*Aaf*)	N (*Aaf*)	F (*Aaf*)
P (*Aaa*)	0.000						
T (*Aaa*)	− 0.019	0.000*					
K (*Aaa*)	− 0.002	0.016	0.000				
G (*Aaa*)	0.002*	− 0.014*	0.001	0.000			
D (*Aaf*)	0.020	0.017	− 0.011	− 0.017			
N (*Aaf*)	− 0.027	0.004*	0.003	− 0.028*	− 0.080		
F (*Aaf*)	− 0.032	0.017*	− 0.003*	0.014	0.023	0.000	
J (*Aaf*)	0.023*	0.041	0.007	− 0.026	0.002	0.045	− 0.031

*Bold values are significant with p value less than 0.05.

### Genetic structure of *Ae. aegypti*

**Figure 5:**
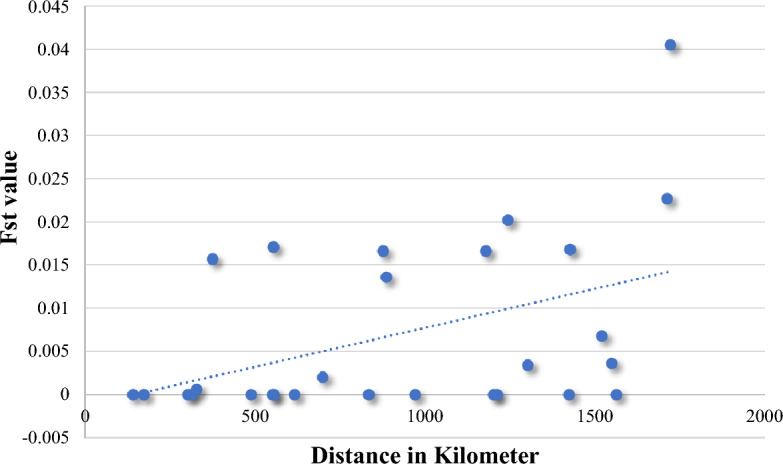
Relationship between pairwise estimates of genetic distance (F_ST_) and geographical distance (km) for *Ae. aegypti* microsatellite data. Trendline shows the general pattern of increasing genetic distance with greater geographic distance (IBD).

**Figure 6:**
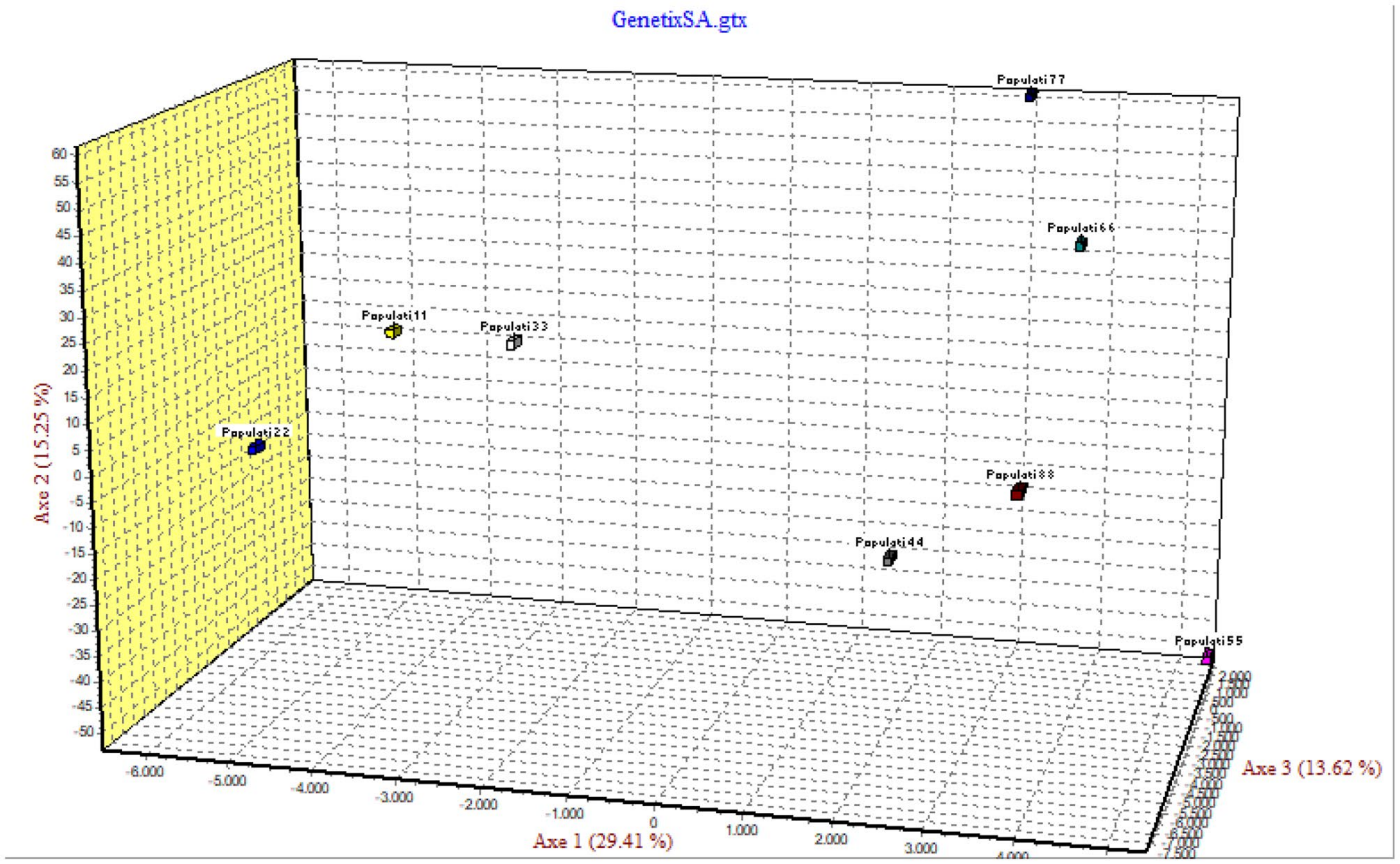
Three-dimensional factorial correspondence analysis (FCA) showing multivariate relationships among eight *Ae. aegypti* populations based on seven microsatellite loci variation. Population 1 Port Sudan, 2 Tokar, 3 Kassala, 4 Barakat/Gezira, 5 Kadugli, 6 Nyala, 7 Fasher, 8 Junaynah.

The unrooted neighbor-joining (NJ) phylogram tree revealed two segregated groups (two main clusters), splitting the localities (Fig. 5). The first group included all three populations of *Aaa* thus Port Sudan, Tokar and Kassala, while the second group contained all populations of *Aaf* thus Kadugli, Nyala, Fasher and Junaynah in addition to Gezira whose *Aaa* clustered in group 2 (Fig. 6).

**Figure 7:**
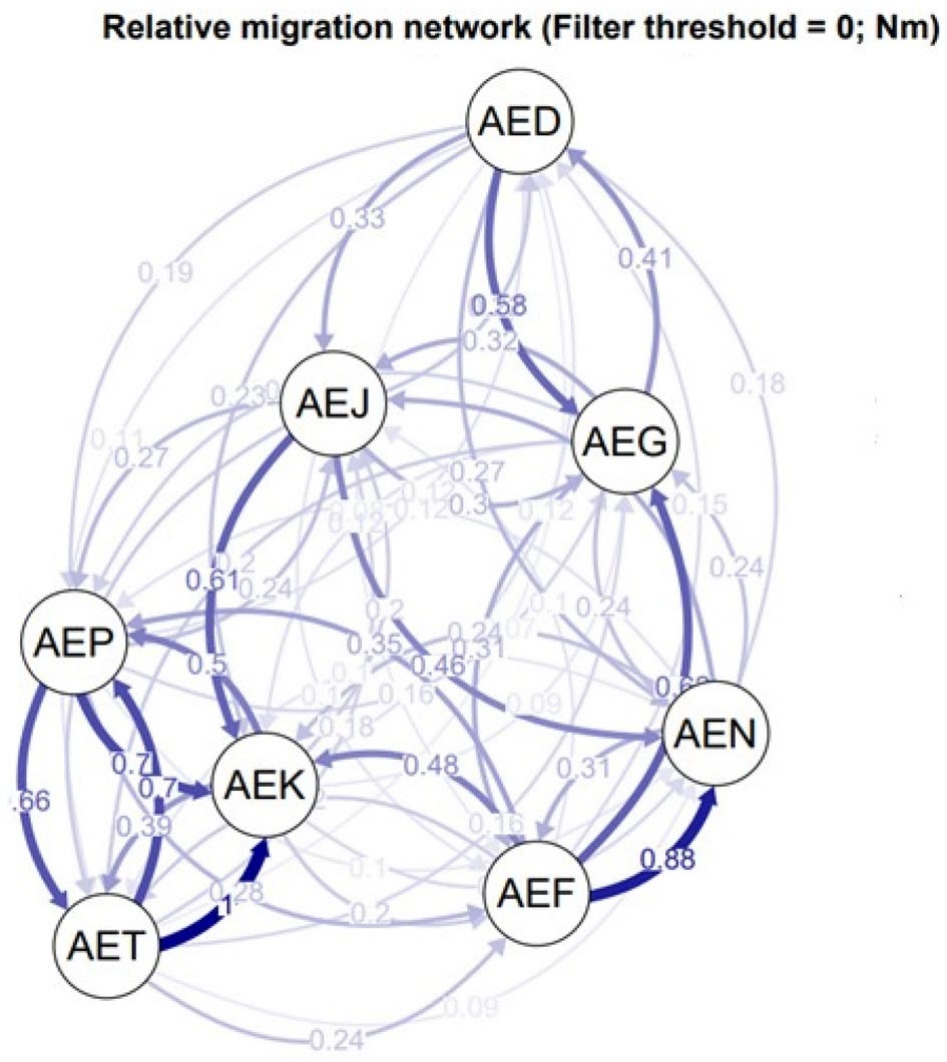
Migration network using divMigrate and based on Nm estimates. Each node represents a population. More gene flow between populations is indicated by the nodes' closeness, and the relative migration values are indicated by the arrows' strong colours. Code for the population names: AED: population from Kadugli, AEJ: population from Junaynah. AEG: population from Barakat/Gezira, AEN: population from Nyala, AEF: population from Fasher, AEP: population from Port Sudan, AEK: population from Kassala, AET: population from Tokar.

The Factorial Correspondence Analysis (FCA) plots suggested that *Aaa* populations from Port Sudan, Tokar and Kassala clustered into one group (which is consistent with the NJ dendrogram tree) while *Aaf* populations of Fasher and Nyala grouped together and Gezira, Kadugli and Junaynah were also revealed to be one group (Fig. 7).

**Figure 8:**
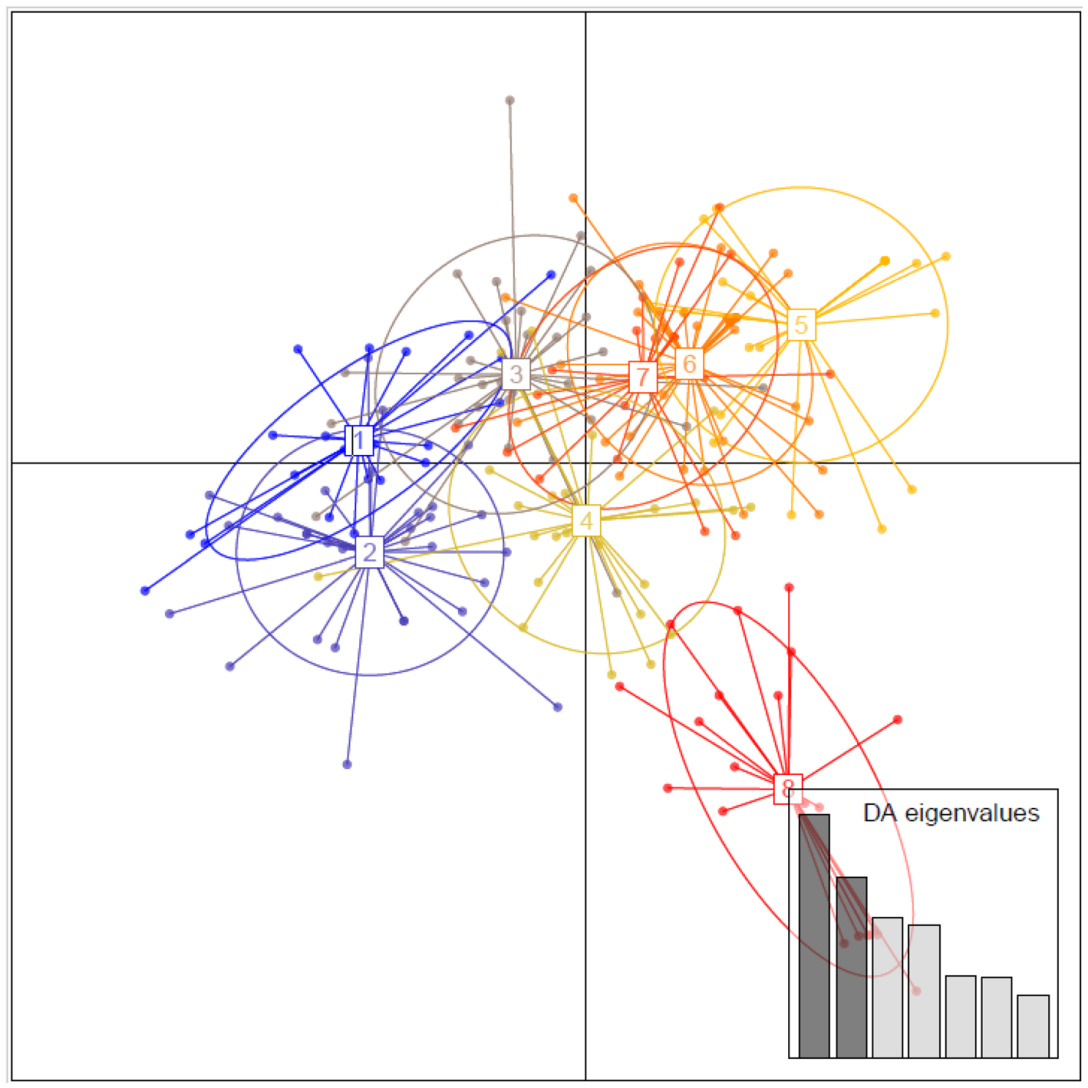
Discriminant Analysis of Principal Components (DAPC) for eight *Aedes aegypti* populations from Sudan using a microsatellite dataset. The graph depicts individuals as dots and groups as inertia ellipses. In the inset, a bar plot of discriminant analysis eigenvalues (DA eigenvalues) is shown. The number of bars represents the number of discriminant functions preserved in the analysis, and the eigenvalues represent the ratio of variance between groups to variation within groups for each discriminant function. 1: Port Sudan; 2: Tokar; 3: Kassala; 4: Barakat/Gezira; 5: Kadugli, 6: Nyala; 7: Fasher; 8: Junaynah.

The Bayesian cluster analysis using STRUCTURE revealed that when K = 2, the estimate of the Delta K and likelihood of the data (LnP(D)) was largest, implying two genetically separate groups (Fig. 7). After completing the first run of Structure + Structure Harvester, group 1 (red cluster) consisted of *Ae. aegypti aegypti* populations east of the Nile River, including Port Sudan, Tokar, Kassala, and Gezira, and group 2 (blue cluster) consisted of *Ae. aegypti formosus* populations Kadugli, Nyala, Fasher, and Junaynah suggesting that there are two main populations (basically, the two subspecies). A similar pattern of population clustering was further substantiated by the DAPC analysis (Fig. 8), where all the populations were seen overlapping, except Junaynah *Aaf* population. However, Kadugli, Nyala and Fasher (*Aaf* populations) appeared to be somewhat distant from the other *Aaa* populations (Fig. 8).

## Discussion

The threat of emerging and re-emerging arboviral infections is quickly increasing over the world, notably in Africa^29^. In Sudan, arboviral illnesses have become a major public health concern. Yellow fever, dengue fever, and chikungunya epidemics have caused substantial mortality and morbidity in different parts of the country during the last two decades, mainly in Port Sudan and Kassala in the east and Darfur in the west^2,5,30,31^. *Aedes aegypti* has been reported in Sudan since 1903 and was described for the first time in Khartoum by Balfour^2^. It plays a critical role in the spread of the viruses that cause these diseases^1,32^.

On the African continent, two *Ae. aegypti* subspecies/forms known as *Ae. aegypti aegypti* (*Aaa*) and *Ae. aegypti formosus* (*Aaf*) exist and these subspecies have differences in their distribution, behaviour, breeding sites, and virus transmission capacity^1,33^. In previous studies conducted by^1,28^, the distribution and genetic diversity of *Aedes aegypti* subspecies across the Sahelian belt in Sudan using the cytochrome oxidase and NADH dehydrogenase subunit 4 (ND4) mitochondrial gene markers were described. In this study, we used microsatellite markers to investigate the genetic structure and differentiation of populations of the *Ae. aegypti* subspecies/forms in Sudan.

Overall, genetic diversity of *Ae. aegypti* estimated in this study was relatively high (NA = 14–37), (AR = 2.8–3.5), (Gd = 0.818–0.915), (HO = 0.878–0.982), (HE = 0.816–0.911) compared to other population structure studies of *Ae. aegypti* using microsatellites. The high alleles number range in this study reflects the vastly polymorphic nature of the selected microsatellites markers. A recent study of Ref.^3^ in *Aedes aegypti* populations in Sudan reported a lower allelic range (7–21) compared to the allelic range reported in this study. However, other research^16,34^ showed an allelic range closer to our study, thus ranging from 15 to 32 and 5 to 36 alleles, respectively. The allelic richness ranged between 4.16 and 8.67 in the study of Ref.^3^ which was higher than the richness in our study (AR = 2.8–3.5) and that of our study was higher than that (1.629 -2.945) of Ref.^34^.

Generally, the populations of *Aaa* possessed slightly higher F_*ST*_ values (F_*ST*_ = 0.023) compared to *Aaf* populations (F_*ST*_ = 0.019) which is consistent with the ND4 MtDNA dataset^28^. However, the CO1 MtDNA genetic diversity revealed contradictory results with no difference between *Aaa* and *Aaf* subspecies^1^. Significant deviations from HWE was revealed in 14 out of 56 tests (25%) and this showed a significant departure from HWE for the two subspecies. All the *Aaa* subspecies/forms’ populations departed from HWE, while in the *Aaf* subspecies/forms, there was a departure in all loci except for A10 and M201. A similar pattern was observed in a study in Senegal that detected HWE in 16 out of 56 possible tests and from those, one significant deviation was detected in the *Aaf* samples and five in *Aaa*^35^. A worldwide study discovered that HWE occurred in 42 of 300 populations of *Ae. aegypti* subspecies/forms populations^22^.

The inbreeding factor revealed a higher average in *Aaa* (F_IS_ average = 0.086) compared to *Aaf* populations (F_IS_ average = 0.077). The average allelic richness showed similarity between the two subspecies populations, which agreed with a study from Gabon and Kenya^18^. The limited linkage disequilibrium was not consistently observed for any locus pair, thus suggesting that linkage disequilibrium was not the result of physical linkage (co-segregation of alleles at loci on the same chromosome). Instead, significant results could most likely be explained by localised demographic effects.

The isolation by distance revealed a highly significant moderate correlation (p = 0.003, correlation coefficient (*r)* = 0.391) between the genetic diversity of the microsatellite genes across the whole populations of *Ae. aegypti* in this study and the geographical distance and this was concordant with^1^ which used CO1 MtDNA dataset resulting in a significant moderate relationship (correlation coefficient value (*r*) = 0.586, *p* = 0.005). Another study in Sudan^3^ also found correlation of genetic variations with the geographical distance between study sites in east and west of Sudan (R^2^ = 0.4272, *p* = 0.01) which strongly supports our finding that the isolation of the subspecies was most probably by distance.

The unrooted neighbor-joining tree clustered the populations of Port Sudan, Tokar and Kassala (*Aaa*) in a group, while Fasher *Aaf* population stood alone, Gezira (*Aaa*) and Kadugli (*Aaf*) clustered together and Junaynah and Nyala (*Aaf*) clustered together, with the exception of Gezira, which clustered with the *Aaf* group, this result is similar to the study of^1^. Also, the genetic structuring of the two subspecies of *Aedes aegypti* is in agreement with the recent study of^3^ which indicated the presence of two genetically distinct subspecies of *Ae. aegypti*.

The three-dimensional factorial correspondence analysis (FCA) plot demonstrated the genetic grouping among sites, Port Sudan, Tokar and Kassala (*Aaa*) grouped together, Fasher and Nyala (*Aaf*) clustered together while Gezira, Kadugli and Junaynah constituted the 3^rd^ group. It is worth noting that members of groups 2 and 3 were geographically related (located in the west and middle parts of the country), while members of group 1 (located in the eastern part of the country) were more diverged (high bootstrap of 95). These results might indicate recent historical gene flow, which could be linked to the geographical distances between the different groups e.g., Port Sudan in group 1 is located only 141 km from Tokar in the same group which is located 1713 km from Junaynah in group 3. The significant relationship noticed in this study in the isolation by distance analysis correlation coefficient (r) = 0.394, p = 0.003 might justify the limited gene flow between the subspecies populations and this has been proven in the migration network which indicated the high gene flow between the geographically closed populations.

AMOVA results of microsatellite genes indicated a high percentage of variance components within populations (96.02%) compared with variation among groups (2.23%). AMOVA results showed higher variation percentages among the two subspecies groups in both mitochondrial genes CO1 and ND4 (39.22% and 26.64% respectively)^1,28^ with high genetic variations within populations (53.53%) and less among groups. Another study^3^ indicated that the majority of genetic variation in *Aedes aegypti* populations from Sudan was among individuals and within regions, with just 5% of the total variation related to variations between groups, which was consistent with this study.

Interestingly, the Bayesian model-based clustering was largely congruent in partitioning the populations into two genetic groups (best structure K = 2) and clearly indicated that the two subspecies/forms populations were structured in two groups. A study conducted in Kenya and Gabon stated comparable conclusions of a first split of all the samples to two clusters (K = 2), however the STRUCTURE results indicate the forms are clearly two, although not totally separated and this split roughly represents the strong genetic differentiation between *Aaf* and *Aaa*, as suggested in previous studies^17,19^. In the case of this study, these two genetically distinct groupings (perhaps linked to the historically documented isolation stated by^36^ and matched with the geographic dispersion reported in^1^, might be attributed.

Although the gene flow across subspecies populations appears low, the Migration network revealed that the gene flow within *Aaa* and *Aaf* populations seemed to be happening according to their geographical location rather than their forms/subspecies. There is a migration and moderate gene flow between Kadugli (*Aaf*) and Gezira (*Aaa*), on the other hand, a strong gene flow was found between the *Aaa* (Port Sudan, Kassala and Tokar) populations. These findings agreed with the study of^1^ which indicated the limited gene flow mostly attributed to the geographical distances as well as different ecological environments restricts the flight range of *Aedes* mosquito and gene flow between the two subspecies populations.

The genetic structure of *Ae. aegypti* subspecies using the microsatellite markers revealed that the populations of the two subspecies were separated as two groups, especially populations of *Aaf* which clustered together while using different clustering methods (NJ tree, FCA plotting and STRUCTURE). Despite the fact that mitochondrial genetic variations^1,28^ revealed low gene flow and high genetic diversity between the two subspecies populations in Sudan, it is difficult to say whether this variation reflects a true difference between the two subspecies or the geographical distances that limited gene flow.

Conversely, a recent study in Gabon and Kenya found little genetic isolation between forest and domestic *Ae. aegypti*, implying that there may be extensive gene flow between them, while phylogenetic relationships revealed a clear separation between the two sites^18^. It is likely that gene flow between the two subspecies began lately, with the *Aaf* invasion into human habitat, where the *Aaa* already existed. Powell and Tabachnick determined from genetic data that there was complete isolation and absence of gene flow between the two subspecies around 400–550 years ago^26^.

In this investigation, the hypothesis defined using microsatellite-based estimates of genetic structure found that the two groups were genetically diverse and distinct. Overall, these findings help us better understand the forms of *Ae. aegypti* in East Africa, where data is scarce. The reality is that various populations have vastly varied vector competencies due to phenotypic variations. The sensitivity of *Ae. aegypti aegypti* to disease transmission may be connected to insect population migration and/or possible intermingling of individuals from different locations. As a result, population genetic studies require determining the genetics of these populations and investigating the genetic variations linked to vector abilities^37^.

Our research explored the genetic structure, gene flow and diversity of the two subspecies of *Aedes aegypti* vector populations in Sudan across different regions. These data can be utilized to track the effectiveness of control measures, changes in gene flow patterns, and new introductions. The vectorial capacity of *Ae. aegypti* populations and subspecies to spread arboviruses varies greatly^33,37,38^.

Bearing in mind that the two subspecies differ in their behaviour and potential to transmit disease, their distribution and existence in each arboviral outbreak area in Sudan should be considered when developing any vector control intervention^1,26,39^. Our findings will be essential to the control program's success if the nation adopts innovative vector control strategies. According to^40^, a genetic alteration that depends on enduring genetic variation in populations must be specific to the intended population.

Other future studies on vector behaviour, vector competence, breeding habitats, genetic variations and structure in other sites using higher sample sizes and study sites and viral transmission of *Ae. aegypti* subspecies vectors are recommended in order to improve the surveillance system of *Ae. aegypti* vector.

Lastly, migrations and mobility caused by humans may promote the long-distance spread of vectors, resulting in the admixture of populations adapted to urban and forest environments, which may have consequences for the management and transmission of disease. The government must designate effective preventive and control measures, increase environmental governance in the areas inhabited by both subspecies in accordance with their vectorial potential and gene flow, and implement mosquito control measures.

## Methods

### Mosquito sample collection and identification

Samples of *Ae. aegypti* larvae and pupae were collected (January 2014- April 2017) from both indoor and outdoor breeding habitats from eight study sites (Port Sudan, Tokar, Kassala, Fasher, Nyala, Gezira, Kadugli, and Junaynah) described in^1^. The study sites were selected according to the past reports of dengue and other arboviruses cases and *Aedes aegypti* vector records. Mosquito aquatic stages were then transferred to the insectarium at National Public Health Laboratory (NPHL) at Khartoum/Sudan where the samples were sorted out, classified, discarded to trays with water and larvae food^1^ and reared to adults at optimum temperature (25 ± 2 °C) and relative humidity (80–90%) with a 12:12 (L: D) photoperiod.

Using appropriate taxonomic keys^41^, the larvae were identified morphologically to their species. After adult emergence, *Ae. aegypti* females were identified to their subspecies according to the morphological taxonomic key^42^. The identified female mosquitoes (*Aaa* and *Aaf*) were individually preserved in labelled microfuge tubes with 70% isopropanol and then placed in a freezer of − 20 °C. The preserved samples were transferred to the Universiti Sains Malaysia (USM) prior to proceeding with the molecular work.

### Genomic DNA extraction

*Aedes aegypti* samples from each study site (a minimum of 10 individuals per site) were used for extraction. Prior to extraction, the mosquito samples were washed twice using ethanol and distilled water and dried out. Using DNeasy Blood and Tissue Extraction Kit (Qiagen, Germany), genomic DNA was extracted from single female mosquitoes following the manufacturer's instructions with minor adjustments (an increase of the incubation time to 65 °C overnight to increase the lyses of the cells). After extraction, genomic DNA was eluted in nuclease-free water and stored in a freezer of − 20 °C. DNA integrity was assessed and visualized on 0.8% (w/v) agarose gel electrophoresis in 0.5X TBE buffer and the quantity was further assessed using UV spectrophotometer Q3000 (Quawell). Species identification was confirmed as explained by^43^.

### Microsatellite DNA molecular technique

Seven microsatellite markers (A10, B07, H08, G11, M313, M201, and B19) designed by^44^ which were single-copy microsatellite sequences identified from enriched plasmid libraries and selected cosmid subclones and have proved quite useful in evaluating the population genetics of *Ae. aegypti* in a number of populations^44^ were selected.

Singleplex PCRs were performed using a BioRad MyCyclerTM Thermal Cycler (BioRad Laboratories, Inc.). According to the supplier’s (Promega Company, USA) reaction mixture guideline, each 50 μl reaction volume contained 10 μl of 5X Green Buffer GoTaq (Promega), 3 μl of 25 mM MgCl_2_, 1 μl of 25 mM dNTP, 1 μl of each primer, 0.25 μl of Taq polymerase, 2 μl (> 50 ng) of template DNA and 31.75 μl of double distilled water. Fluorescent (two dyes) primers were used due to the further fragment analysis as shown in Table 8.

**Table 8:** Characteristics of seven polymorphic microsatellite loci *Ae. aegypti*.

Locus	Repeat motif	Primer 5'-3'	Accession number	Allele size range (bp)	Temp (°C)	Fluorescent dye
A10	CT_10_ (TT) CT	F-AATCGTGACGCGTCTTTTG	DU169901	233–239	53.7 °C	FAM
		R-TAACTGCATCGAGGGAAACC				
B07	GA_15_	F-CAAACAACGAACTGCTCACG	DU169902	157–183	54.8 °C	FAM
		R-TCGCAATTTCAACAGGTAGG				
H08	TCG_7_	F-AAAAACCACGATCACCGAAG	DU169903	199–205	53.4 °C	FAM
		R-ACGCGATCACACACTGAAAATG				
G11	TTA_16_	F-TGTCTCATGGATTGCCTTATT	DU169906	240–300	51.7 °C	FAM
		R-GTCAGAACTTTTGGGGACCA				
B19	CAT_7_	F-ATTGGCGTGAGAACATTTTG	DU169905	156–186	51.7 °C	FAM
		R-GAGGAGTGAGCAGATAGGAGTG				
M313	ATG_5_(ATA)ATG	F-CACCTCGTGACATACAAACACC	DU169909	117–123	55.9 °C	HEX
		R-ACGTACCCAAGCCACGTACA				
M201	ATA_36_	F-GGAGCATTCATAGAGAATTGTCA	DU1635091	110–116	54.1 °C	FAM
		R-GAGATGAACCAGTCATAGGGC				

The PCR cycling conditions were initial denaturation of 94 °C for 5 min, 30 cycles of 94 °C for 1 min, 60 °C annealing temperature for 1 min and extension at 72 °C for 2 min with a 10 min final extension at 72 °C for the marker primers A10, B07, H08 and G11. The cycling conditions of initial denaturation 94 °C for 5 min followed by 39 cycles of 94 °C for 20 s, annealing temperature of 55 °C for 20 s and an extension of 72 °C for 30 s and final extension at 72 °C for 10 min for B19, M313 and M201 marker primers. Primers, annealing temperatures, and their sequences are presented in Table 8.

The PCR products were analyzed in agarose gel electrophoresis of 2% and visualized under ultraviolet light using GelDoc-It^®^ TS 310 UV documentation System (Ultraviolet Products Ltd. Cambridge, UK). Samples with clear bands were sent to NHK Bioscience Solutions Sdn. Bhd for fragment analyses using Applied Biosystems 3730XL DNA Analyzer.

### Standard genetic procedures and variability

Peak size of each individual microsatellite allele fragment was identified, analysed, and scored using Peak Scanner v1.0 (Applied Biosystems) with internal size standard (GS500LIZ). Samples were rescored, and amplification procedures (if possible) repeated, whenever PCR irregularities were encountered. Allele peaks in the electrophoretogram were scored according to^45^. MicroChecker v2.2.3^46^ was used to identify and rectify data irregularities including typographic errors, scoring errors due to dropout of broad alleles or stutter peaks due to low DNA quality and detect and correct microsatellite null alleles.

CONVERT v1.31^47^ and PGDSpider v2.1.0.3^48^ were used to create summary statistics for microsatellite data (allele frequencies and private allele for each locus in each population) and also used to convert the raw data so that it could be analysed in various software packages^47,48^.

### HWE, LD and FIS estimations

Arlequin v3.5.2.2^49^ was used for genetic variation assessment through measuring mean of both allele numbers (NA) per locus and population and allele size range. The software package FSTAT v2.9.3.2^50^ was used to analyse diversity among sites using allelic richness (AR) (with the rarefaction method to correct for differences in sample size). Using Arlequin v3.5.2.2^49^, we estimated observed (HO) and expected (HE) heterozygosity per locus and population, as well as mean genetic heterozygosity across all loci. Using the same program, the deviation from Hardy–Weinberg Equilibrium (HWE) was calculated based on exact testing with 10,000 Markov chain stages and 5000 dememorization steps. The likelihood ratio test of linkage disequilibrium based on the Expectation–Maximization (EM) algorithm^51^ was performed on all pairwise locus comparisons for all sites in Arlequin v3.5.2.2^49^ with 10,000 permutations to test for the presence of significant association between alleles among loci pairs. With 10,000 permutations, an exact test was performed to look for statistically significant deviations from independent segregation of genotypes linkage equilibrium [linkage disequilibrium (LD)], followed by the false discovery rate (FDR) adjustment^52^ at the 9% significance level. The inbreeding coefficient (F_IS_) was also estimated using the software program FSTAT Version 2.9.3.2^50^, with a value ranging from − 1 (no inbreeding) to + 1 (high inbreeding) (total identical).

In order to determine occurrence of recent effective population size reduction, BOTTLENECK v1.2.02^53,54^ was used to perform Wilcoxon sign-rank test and mode shift test (distortion of the typical L- shape distribution). Wilcoxon's test was run using the two-phased mutation model (TPM)^53,55^ setting the proportion of Stepwise mutation model (SMM) in respect to TPM to 95% and the variance to 12. A total of 5000 simulation iterations was conducted, as suggested by^54^. This included 95 percent single stepwise mutation and 5% infinite allele mutation with statistical significance determined using 1000 simulations.

### Genetic structure and variations

An unrooted Neighbour-Joining phylogenetic tree was created with POPTREE2^56^ using Nei's genetic distance (DA)^57^ and 1000 bootstrap replications to determine the confidence level of each node to visualize the relationships among sites^58^. Pairwise genetic divergence values between populations were estimated in Arlequin v3.5.2.2^49^ using F_*ST*_ (proportion of the total genetic variance contained in a subpopulation (the S subscript) relative to the total genetic variance (the T subscript) values. The possible values are 0 to 1. A high F_*ST*_ suggests that populations differ significantly from one another, with statistical significance based on 10,000 permutations. Different hierarchical Analyses of Molecular Variance (AMOVA) to evaluate the relative attribution of variance among populations, among individuals within populations, and within individuals with 1000 random permutations was used to perform hierarchical variation structuring in Arlequin v3.5.2.2^49^.

The Mantel correlation coefficient (r) between matrices of genetic (F_*ST*_) and geographic distance was calculated using Arlequin v3.5.2.2^49^ with 10,000 random permutations to see if genetic relationships among sampling areas conformed to a pattern of genetic isolation by distance (IBD). Microsoft Excel was used to create isolation by distance charts (km).

The Factorial Correspondence Analysis (FCA) was done as a complementary approach to a univariate test like F_*ST*_ since multilocus population genetic data are multivariate in nature^59^. It was employed to assess population subdivision on pairwise genetic distance among 202 individuals from eight *Ae. aegypti* populations.

GENETIX version 4.05^60^ was used to perform FCA based on genotypic data obtained for individuals from the populations. Correction for multiple testing for HWE, LD, FIS and Wilcoxon’s test was performed using the FDR approach as described in Benjamini & Hochberg (1995) at the 95% confidence level. Additionally, a clustering analysis was performed using Discriminant Analysis on Principal Components (DAPC) from Adegenet (Jombart, 2008). Furthermore, divMigrate (https://popgen.shinyapps.io/divMigrate-online/) was used to construct a network representing the relative rate and direction of migration among populations^61^, with Nm as the measure of genetic distance. The significance of the Nm values was determined by performing 1000 bootstraps with = 0.05.

The hierarchical variations could be attributed to differences between groups; according to the subspecies populations, the subspecies identified in Ref.^28^ as well as clustering according to K = 2 from STRUCTURE were estimated. Three hierarchical levels of variation were tested for each run, among groups within total (F_CT_), among populations within groups (F_SC_) and among populations within total (F_*ST*_).

Following that, two distinct clustering methods were employed to identify groups of genetically related individuals and sampling locations, as well as to assess their spatial distribution. First, individuals were assigned to clusters using a Bayesian model-based clustering approach performed in STRUCTURE v2.3.3^62^. The Bayesian clustering methodology employed in Structure 2.3.4 offered a comparative assessment of population structure^62,63^. The number of clusters (K) was calculated using the web software Structure Harvester, as reported by Ref.^64^. Using the online software Structure Harvester, after performing 15 independent runs of K = 1 to 8 at 10,000 Markov Chain Monte-Carlo (MCMC) repetitions and a burn‐in period of 1000 iterations, Admixture model and correlated allele frequencies were utilized, together with a uniform prior for α, with an initial value of 1 and maximum of 10.0; λ was set at 1.0. For the selected value of K, we assessed the membership coefficients per individual per cluster (Q), setting the assignment threshold to Q > 0.80. Using STRUCTURE Harvester v0.6.94, the best number of clusters was illustrated by plotting the average estimated LnP(D) (Ln probability of the data) and the K technique of Ref.^64^.

## Conclusion

While understanding the genetic variety and composition of disease vectors is crucial for managing them, this information is frequently inadequate. The two groupings that resulted from the analysis of the populations were suggested to be two genetically different groups (subspecies). Geographical distances and genetic variation showed a moderate to strong significant association. Subspecies populations appear to migrate and exchange genes based on their geographic proximity. In Sudan and other African nations, when it comes to the spread of dengue disease, chikungunya, yellow fever, and other arboviruses, research is required to comprehend the ecological factors that influence the distribution and transmission capacity of the two subspecies and to create effective viral control initiatives.

### Supplementary Information

Supplementary Information.

## Data Availability

All data generated or analysed during this study are included in this published article [Supplementary Files].
