# Supplementary material for: Population genetic structure of Aedes aegypti subspecies in selected geographical locations in Sudan
Source: Sci Rep. 2024 Feb 5;14:2978. doi: 10.1038/s41598-024-52591-6 (PMC10844603; doi:10.1038/s41598-024-52591-6)
Supplement: Supplementary file 1 — Supplementary Information. [file 41598_2024_52591_MOESM1_ESM.docx]

**Supplementary 1:** Scoring information of seven nuclear microsatellites markers in eight *Aedes aegypti* populations from Sudan:

|  | **A10** |  | **B07** |  | **H08** |  | **G11** |  | **B19** |  | **M313** |  | **M201** |  |
| --- | --- | --- | --- | --- | --- | --- | --- | --- | --- | --- | --- | --- | --- | --- |
| Pop = Port Sudan |  |  |  |  |  |  |  |  |  |  |  |  |  |  |
| P01 | ? | ? | 170 | 172 | 200 | 204 | ? | ? | 175 | 181 | 115 | 123 | 111 | 113 |
| P02 | ? | ? | 176 | 169 | 204 | 204 | 294 | 295 | 182 | 185 | 117 | 124 | 110 | 112 |
| P03 | 235 | 236 | 157 | 181 | 201 | 204 | ? | ? | 175 | 181 | 118 | 125 | ? | ? |
| P04 | 235 | 236 | 156 | 171 | 201 | 207 | 259 | 259 | 179 | 182 | 118 | 124 | ? | ? |
| P05 | ? | ? | 155 | 170 | 201 | 206 | ? | ? | 176 | 179 | 118 | 124 | 110 | 112 |
| P06 | 236 | 238 | 168 | 172 | 204 | 205 | 259 | 260 | 176 | 179 | 117 | 125 | 105 | 112 |
| P07 | ? | ? | 175 | 179 | 204 | 204 | ? | ? | 175 | 179 | 118 | 121 | 112 | 113 |
| P08 | 235 | 237 | 176 | 178 | 201 | 204 | 259 | 260 | 159 | 181 | 112 | 126 | 112 | 113 |
| P09 | ? | ? | 158 | 163 | 201 | 204 | 258 | 259 | 156 | 172 | ? | ? | 108 | 111 |
| P10 | 236 | 238 | 159 | 181 | 201 | 204 | 256 | 259 | 178 | 181 | ? | ? | ? | ? |
| P11 | 238 | 239 | 160 | 164 | 201 | 204 | 256 | 258 | 179 | 182 | 113 | 121 | 106 | 113 |
| P12 | 238 | 239 | 164 | 170 | 201 | 204 | 256 | 259 | 180 | 183 | 114 | 124 | 106 | 113 |
| P13 | 236 | 239 | 172 | 178 | 201 | 204 | ? | ? | 164 | 170 | ? | ? | ? | ? |
| P14 | 235 | 236 | 159 | 165 | 201 | 204 | 256 | 260 | 177 | 180 | 116 | 117 | 108 | 111 |
| P15 | ? | ? | 176 | 179 | 201 | 207 | ? | ? | 176 | 179 | ? | ? | ? | ? |
| P16 | 236 | 238 | 156 | 164 | 200 | 204 | ? | ? | 176 | 182 | 118 | 121 | 106 | 113 |
| P17 | 236 | 238 | 174 | 178 | 204 | 207 | 259 | 259 | 179 | 182 | 118 | 124 | 105 | 112 |
| P18 | 231 | 238 | 171 | 178 | 200 | 203 | ? | ? | 160 | 181 | ? | ? | ? | ? |
| P19 | 231 | 238 | 164 | 174 | 200 | 213 | 295 | 295 | 179 | 182 | 118 | 121 | 111 | 113 |
| P20 | 236 | 238 | 167 | 171 | 204 | 205 | 256 | 260 | 167 | 171 | ? | ? | ? | ? |
| P21 | 234 | 236 | 175 | 181 | 198 | 204 | ? | ? | 178 | 184 | ? | ? | 110 | 112 |
| pop = Tokar |  |  |  |  |  |  |  |  |  |  |  |  |  |  |
| T01 | 232 | 236 | 169 | 179 | ? | ? | ? | ? | ? | ? | ? | ? | ? | ? |
| T02 | 232 | 236 | ? | ? | ? | ? | ? | ? | 179 | 179 | 121 | 121 | 106 | 112 |
| T03 | 234 | 238 | 173 | 175 | ? | ? | ? | ? | 176 | 187 | 118 | 124 | 108 | 112 |
| T04 | 232 | 238 | 164 | 173 | ? | ? | ? | ? | 179 | 182 | 118 | 124 | 106 | 113 |
| T05 | 233 | 238 | 169 | 173 | ? | ? | 259 | 260 | 178 | 183 | ? | ? | 108 | 111 |
| T06 | 236 | 239 | 164 | 174 | 204 | 205 | 259 | 260 | 175 | 181 | 117 | 124 | 110 | 111 |
| T07 | 234 | 236 | 165 | 169 | 201 | 204 | 256 | 259 | 176 | 179 | 118 | 124 | 112 | 113 |
| T08 | 232 | 236 | 165 | 169 | ? | ? | 294 | 296 | 175 | 181 | ? | ? | ? | ? |
| T09 | 233 | 238 | 154 | 158 | 198 | 201 | 259 | 260 | 179 | 182 | 118 | 124 | 108 | 111 |
| T10 | ? | ? | 159 | 173 | 204 | 207 | 256 | 259 | 154 | 158 | ? | ? | ? | ? |
| T11 | ? | ? | 153 | 158 | 201 | 204 | 258 | 259 | ? | ? | ? | ? | ? | ? |
| T12 | ? | ? | 153 | 159 | 201 | 204 | 258 | 259 | ? | ? | ? | ? | ? | ? |
| T13 | ? | ? | 153 | 159 | 204 | 207 | 258 | 259 | ? | ? | ? | ? | ? | ? |
| T14 | 235 | 237 | 164 | 172 | 198 | 204 | 258 | 259 | ? | ? | ? | ? | ? | ? |
| T15 | ? | ? | 153 | 159 | 201 | 207 | ? | ? | ? | ? | ? | ? | 110 | 111 |
| T16 | ? | ? | 159 | 159 | 204 | 206 | 259 | 261 | ? | ? | ? | ? | ? | ? |
| T17 | ? | ? | 153 | 159 | 201 | 204 | 256 | 259 | ? | ? | ? | ? | ? | ? |
| T18 | ? | ? | 153 | 159 | 201 | 204 | 256 | 259 | ? | ? | ? | ? | ? | ? |
| T19 | 231 | 236 | 153 | 159 | 201 | 207 | ? | ? | ? | ? | ? | ? | ? | ? |
| T20 | 235 | 236 | 171 | 175 | 205 | 207 | ? | ? | ? | ? | ? | ? | ? | ? |
| T21 | 236 | 238 | 167 | 171 | 201 | 204 | 258 | 259 | ? | ? | ? | ? | ? | ? |
| T22 | 236 | 238 | 167 | 171 | 201 | 204 | 258 | 259 | 158 | 158 | ? | ? | 108 | 111 |
| T23 | 230 | 236 | 165 | 177 | 198 | 204 | ? | ? | 173 | 176 | 124 | 124 | 108 | 111 |
| T24 | 232 | 238 | 173 | 176 | 201 | 207 | ? | ? | 157 | 176 | 118 | 125 | 109 | 111 |
| T25 | 230 | 236 | 169 | 171 | 198 | 203 | ? | ? | 173 | 176 | 118 | 125 | ? | ? |
| T26 | 230 | 234 | ? | ? | 204 | 207 | 259 | 260 | 173 | 182 | 117 | 123 | 108 | 112 |
| T27 | 232 | 238 | 165 | 171 | ? | ? | ? | ? | 176 | 179 | 118 | 124 | 112 | 117 |
| T28 | 232 | 239 | 165 | 175 | 201 | 204 | 258 | 259 | 179 | 182 | 118 | 118 | 108 | 111 |
| T29 | 232 | 236 | 169 | 175 | 201 | 207 | ? | ? | 176 | 182 | 118 | 125 | 107 | 111 |
| T30 | 231 | 236 | 169 | 173 | 204 | 206 | ? | ? | 176 | 179 | 118 | 124 | 108 | 111 |
| T31 | 232 | 239 | 171 | 175 | 201 | 204 | 259 | 260 | 176 | 179 | 118 | 121 | 111 | 117 |
| pop= Kassala |  |  |  |  |  |  |  |  |  |  |  |  |  |  |
| K01 | 232 | 238 | ? | ? | ? | ? | ? | ? | 168 | 183 | ? | ? | 107 | 113 |
| K02 | ? | ? | 155 | 160 | 199 | 206 | ? | ? | 154 | 160 | ? | ? | ? | ? |
| K03 | 227 | 230 | 163 | 163 | 203 | 207 | 256 | 258 | ? | ? | ? | ? | ? | ? |
| K04 | 227 | 233 | 163 | 173 | 200 | 206 | 259 | 260 | 176 | 179 | 113 | 127 | 106 | 113 |
| K05 | 236 | 238 | 167 | 171 | 204 | 207 | ? | ? | ? | ? | 123 | 123 | ? | ? |
| K06 | ? | ? | 162 | 162 | 198 | 201 | ? | ? | 176 | 182 | 117 | 124 | 109 | 112 |
| K07 | ? | ? | 168 | 177 | 199 | 206 | ? | ? | ? | ? | 118 | 121 | ? | ? |
| K08 | 232 | 236 | 169 | 173 | 201 | 204 | 259 | 260 | 168 | 177 | 116 | 126 | ? | ? |
| K09 | 230 | 236 | 167 | 173 | 201 | 207 | ? | ? | ? | ? | ? | ? | 108 | 113 |
| K10 | 229 | 231 | 169 | 171 | 201 | 207 | 259 | 260 | 175 | 178 | 120 | 126 | 112 | 113 |
| K11 | ? | ? | 153 | 159 | 201 | 204 | 259 | 260 | ? | ? | ? | ? | 112 | 113 |
| K12 | 233 | 238 | 159 | 159 | 201 | 204 | 272 | 272 | ? | ? | ? | ? | ? | ? |
| K13 | ? | ? | 173 | 176 | 201 | 204 | ? | ? | 158 | 160 | 117 | 121 | 104 | 111 |
| K14 | ? | ? | 168 | 170 | 198 | 204 | ? | ? | ? | ? | ? | ? | ? | ? |
| K15 | 231 | 238 | 170 | 178 | 201 | 207 | ? | ? | 179 | 182 | ? | ? | 112 | 113 |
| K16 | 233 | 236 | 176 | 182 | 202 | 205 | 284 | 294 | 179 | 185 | 121 | 127 | 106 | 111 |
| K17 | ? | ? | 173 | 177 | ? | ? | ? | ? | 181 | 184 | 116 | 123 | 106 | 112 |
| K18 | 231 | 238 | 168 | 170 | ? | ? | ? | ? | 169 | 171 | 116 | 122 | 108 | 113 |
| K19 | ? | ? | 179 | 185 | 206 | 206 | 281 | 283 | 179 | 184 | 118 | 124 | 110 | 116 |
| K20 | 231 | 238 | 174 | 178 | 204 | 207 | ? | ? | 166 | 175 | 118 | 125 | 111 | 116 |
| K21 | ? | ? | 173 | 180 | 201 | 207 | ? | ? | 158 | 168 | 116 | 123 | 108 | 111 |
| K22 | ? | ? | 162 | 164 | 203 | 207 | ? | ? | 158 | 164 | ? | ? | 111 | 116 |
| K23 | ? | ? | 160 | 165 | 201 | 207 | 259 | 261 | 157 | 160 | 118 | 123 | 106 | 110 |
| K24 | 231 | 238 | 176 | 184 | 201 | 204 | 259 | 259 | 173 | 179 | 118 | 123 | 109 | 111 |
| K25 | 231 | 238 | 164 | 172 | 201 | 204 | ? | ? | ? | ? | 119 | 129 | 106 | 113 |
| K26 | 231 | 238 | 162 | 178 | 201 | 204 | ? | ? | 159 | 159 | ? | ? | 105 | 111 |
| K27 | ? | ? | 160 | 165 | 201 | 207 | 259 | 260 | 164 | 172 | ? | ? | ? | ? |
| K28 | 231 | 240 | 159 | 159 | 201 | 207 | ? | ? | 159 | 159 | ? | ? | ? | ? |
| K29 | 231 | 238 | 162 | 170 | 201 | 204 | ? | ? | ? | ? | ? | ? | ? | ? |
| K30 | 238 | 240 | 164 | 178 | 204 | 207 | 259 | 260 | ? | ? | ? | ? | ? | ? |
| pop= Gezira |  |  |  |  |  |  |  |  |  |  |  |  |  |  |
| G01 | ? | ? | 163 | 177 | 194 | 195 | ? | ? | 163 | 177 | 117 | 124 | 111 | 116 |
| G02 | 233 | 236 | 163 | 173 | ? | ? | ? | ? | ? | ? | 118 | 124 | ? | ? |
| G03 | 231 | 234 | 162 | 170 | 195 | 197 | 296 | 297 | 152 | 158 | 118 | 128 | 108 | 111 |
| G04 | 231 | 236 | 170 | 170 | 205 | 209 | 293 | 295 | 157 | 183 | ? | ? | 105 | 112 |
| G05 | 232 | 234 | 169 | 173 | ? | ? | ? | ? | 176 | 182 | 118 | 125 | 110 | 116 |
| G06 | 237 | 237 | 174 | 174 | ? | ? | ? | ? | 176 | 176 | 118 | 124 | 108 | 111 |
| G07 | 234 | 234 | 173 | 174 | 207 | 207 | ? | ? | ? | ? | 119 | 124 | 113 | 114 |
| G08 | 230 | 232 | 161 | 171 | 191 | 194 | ? | ? | 158 | 158 | 118 | 128 | 108 | 111 |
| G09 | 237 | 239 | 171 | 175 | 191 | 191 | 294 | 295 | 182 | 185 | 118 | 128 | ? | ? |
| G10 | 232 | 238 | 177 | 177 | 198 | 207 | ? | ? | ? | ? | 116 | 122 | 110 | 112 |
| G11 | 229 | 231 | 160 | 166 | 201 | 204 | ? | ? | 180 | 180 | ? | ? | ? | ? |
| G12 | 232 | 234 | 169 | 171 | 198 | 204 | ? | ? | 179 | 184 | 114 | 117 | 107 | 113 |
| G13 | 232 | 238 | 175 | 181 | 201 | 204 | ? | ? | 158 | 158 | 118 | 125 | ? | ? |
| G14 | 230 | 236 | 169 | 181 | 202 | 205 | ? | ? | 173 | 176 | 118 | 124 | 105 | 111 |
| G15 | 235 | 239 | 168 | 173 | 198 | 201 | ? | ? | 155 | 176 | 118 | 125 | 108 | 113 |
| G16 | 230 | 236 | 158 | 185 | 201 | 204 | 266 | 269 | 156 | 176 | 118 | 124 | 108 | 111 |
| G17 | 233 | 240 | 164 | 178 | 198 | 204 | ? | ? | ? | ? | 119 | 119 | 113 | 116 |
| G18 | 232 | 238 | 176 | 184 | 201 | 204 | ? | ? | 153 | 154 | 118 | 123 | 110 | 113 |
| G19 | 232 | 236 | 155 | 170 | 201 | 203 | ? | ? | ? | ? | ? | ? | ? | ? |
| G20 | 230 | 232 | 167 | 173 | 201 | 204 | ? | ? | 155 | 176 | 117 | 117 | ? | ? |
| G21 | 232 | 236 | 161 | 179 | 201 | 208 | ? | ? | ? | ? | ? | ? | ? | ? |
| G22 | 230 | 236 | ? | ? | 201 | 207 | ? | ? | 156 | 156 | ? | ? | ? | ? |
| G23 | 232 | 238 | 165 | 173 | 203 | 203 | 299 | 301 | 177 | 186 | 118 | 124 | 113 | 113 |
| G24 | ? | ? | 157 | 162 | 201 | 208 | ? | ? | ? | ? | ? | ? | ? | ? |
| G25 | 230 | 236 | 165 | 179 | 202 | 205 | ? | ? | 179 | 182 | ? | ? | 104 | 104 |
| pop = Kadugli |  |  |  |  |  |  |  |  |  |  |  |  |  |  |
| D01 | 229 | 237 | 164 | 166 | ? | ? | ? | ? | ? | ? | 118 | 125 | 109 | 113 |
| D02 | 231 | 237 | 168 | 172 | 209 | 213 | ? | ? | 182 | 185 | 118 | 124 | ? | ? |
| D03 | 228 | 230 | 163 | 172 | 195 | 197 | 295 | 299 | 157 | 185 | 118 | 124 | ? | ? |
| D04 | 230 | 232 | 171 | 179 | 204 | 206 | 295 | 300 | 173 | 185 | 116 | 127 | ? | ? |
| D05 | 232 | 238 | 159 | 172 | 201 | 203 | 286 | 287 | 173 | 185 | 118 | 126 | 107 | 112 |
| D06 | 230 | 233 | 166 | 173 | 200 | 203 | 293 | 294 | 160 | 161 | 118 | 124 | 105 | 112 |
| D07 | 230 | 231 | 160 | 168 | 203 | 207 | 286 | 291 | 168 | 173 | 118 | 124 | 107 | 112 |
| D08 | 229 | 230 | 160 | 167 | 202 | 205 | 281 | 297 | 176 | 186 | 118 | 124 | 101 | 103 |
| D09 | 231 | 235 | 168 | 174 | 208 | 209 | 294 | 299 | 160 | 161 | 118 | 128 | 112 | 117 |
| D10 | 232 | 236 | 169 | 175 | 201 | 203 | 307 | 308 | 156 | 174 | 118 | 124 | ? | ? |
| D11 | 232 | 233 | 157 | 164 | 198 | 201 | 295 | 298 | 166 | 185 | 118 | 125 | 108 | 112 |
| D12 | 232 | 236 | 159 | 171 | 198 | 201 | ? | ? | 160 | 167 | ? | ? | ? | ? |
| D13 | 231 | 235 | 172 | 180 | 201 | 204 | ? | ? | 166 | 170 | ? | ? | ? | ? |
| D14 | 232 | 238 | 166 | 173 | 201 | 204 | 299 | 299 | 161 | 174 | 118 | 120 | ? | ? |
| D15 | 228 | 234 | 171 | 173 | 201 | 204 | 294 | 295 | 157 | 184 | 118 | 128 | 107 | 112 |
| D16 | 231 | 235 | 157 | 158 | 198 | 204 | 294 | 295 | 185 | 186 | 118 | 125 | 112 | 112 |
| D17 | 232 | 234 | 161 | 167 | 201 | 204 | ? | ? | ? | ? | ? | ? | ? | ? |
| D18 | 231 | 236 | 160 | 168 | 201 | 204 | ? | ? | 160 | 162 | ? | ? | ? | ? |
| D19 | 233 | 238 | 168 | 171 | 201 | 204 | ? | ? | 157 | 175 | ? | ? | ? | ? |
| D20 | 233 | 234 | 160 | 176 | 204 | 207 | 245 | 248 | ? | ? | ? | ? | ? | ? |
| D21 | 230 | 236 | 155 | 169 | ? | ? | ? | ? | 157 | 158 | 116 | 122 | ? | ? |
| D22 | 234 | 238 | ? | ? | ? | ? | ? | ? | 158 | 158 | 116 | 122 | ? | ? |
| pop = Nyala |  |  |  |  |  |  |  |  |  |  |  |  |  |  |
| N01 | 230 | 238 | 156 | 161 | ? | ? | ? | ? | 156 | 161 | 115 | 126 | ? | ? |
| N02 | ? | ? | 151 | 156 | ? | ? | ? | ? | 171 | 174 | 118 | 130 | ? | ? |
| N03 | 231 | 236 | 160 | 182 | ? | ? | 290 | 303 | 158 | 161 | 118 | 124 | 102 | 110 |
| N04 | 232 | 234 | 167 | 171 | 198 | 204 | ? | ? | ? | ? | ? | ? | 111 | 116 |
| N05 | ? | ? | ? | ? | 200 | 203 | ? | ? | 179 | 182 | 118 | 123 | 109 | 110 |
| N06 | ? | ? | 156 | 164 | 205 | 208 | ? | ? | ? | ? | 117 | 124 | 111 | 113 |
| N07 | 231 | 236 | 164 | 165 | 200 | 203 | ? | ? | ? | ? | ? | ? | 111 | 115 |
| N08 | ? | ? | 164 | 174 | 197 | 203 | ? | ? | ? | ? | 123 | 123 | 110 | 111 |
| N09 | ? | ? | 157 | 164 | 198 | 200 | ? | ? | ? | ? | ? | ? | ? | ? |
| N10 | ? | ? | 156 | 176 | 200 | 206 | ? | ? | ? | ? | ? | ? | 111 | 112 |
| N11 | 230 | 231 | 167 | 169 | 200 | 203 | 294 | 299 | 164 | 178 | 118 | 124 | 108 | 112 |
| N12 | 232 | 233 | 157 | 185 | 198 | 204 | ? | ? | 172 | 175 | 118 | 123 | 110 | 111 |
| N13 | ? | ? | 158 | 165 | 192 | 200 | ? | ? | 156 | 180 | 117 | 124 | 106 | 109 |
| N14 | ? | ? | 167 | 171 | ? | ? | ? | ? | 156 | 179 | 118 | 124 | 105 | 110 |
| N15 | 230 | 234 | 156 | 161 | ? | ? | ? | ? | 184 | 186 | 118 | 128 | ? | ? |
| N16 | 231 | 231 | 161 | 161 | ? | ? | ? | ? | ? | ? | ? | ? | ? | ? |
| N17 | 231 | 233 | 169 | 170 | 201 | 204 | ? | ? | ? | ? | ? | ? | ? | ? |
| N18 | ? | ? | 162 | 170 | ? | ? | ? | ? | 185 | 188 | 118 | 124 | 104 | 110 |
| N19 | ? | ? | ? | ? | ? | ? | ? | ? | 171 | 174 | 118 | 124 | ? | ? |
| N20 | 229 | 231 | 170 | 178 | ? | ? | 280 | 283 | 182 | 185 | 118 | 124 | ? | ? |
| N21 | 237 | 238 | 157 | 166 | ? | ? | ? | ? | 167 | 173 | 118 | 125 | 110 | 113 |
| N22 | ? | ? | 155 | 156 | 197 | 201 | 255 | 267 | 185 | 186 | 118 | 127 | 108 | 110 |
| N23 | 232 | 233 | 156 | 163 | ? | ? | ? | ? | 173 | 184 | 118 | 124 | 112 | 113 |
| N24 | 236 | 236 | 169 | 169 | 201 | 204 | ? | ? | 176 | 186 | 118 | 124 | ? | ? |
| N25 | 229 | 232 | 161 | 161 | ? | ? | 273 | 273 | 179 | 182 | 118 | 125 | 109 | 111 |
| N26 | ? | ? | 169 | 173 | 203 | 206 | ? | ? | 167 | 173 | 118 | 127 | ? | ? |
| N27 | 227 | 231 | 158 | 165 | 198 | 204 | ? | ? | 178 | 181 | 117 | 124 | 109 | 116 |
| N28 | ? | ? | 156 | 173 | ? | ? | ? | ? | 178 | 182 | 117 | 124 | 109 | 116 |
| N29 | ? | ? | 182 | 185 | 213 | 216 | 268 | 294 | ? | ? | 118 | 125 | 110 | 112 |
| N30 | 230 | 234 | 160 | 163 | ? | ? | ? | ? | ? | ? | ? | ? | 113 | 113 |
| N31 | 229 | 231 | 177 | 182 | ? | ? | ? | ? | 160 | 174 | 118 | 125 | ? | ? |
| N32 | ? | ? | 158 | 165 | 201 | 204 | ? | ? | ? | ? | ? | ? | ? | ? |
| N33 | 238 | 240 | 176 | 178 | ? | ? | ? | ? | ? | ? | ? | ? | ? | ? |
| pop= Fasher |  |  |  |  |  |  |  |  |  |  |  |  |  |  |
| F01 | 229 | 231 | 162 | 176 | 201 | 204 | ? | ? | ? | ? | ? | ? | ? | ? |
| F02 | 235 | 238 | 157 | 173 | 198 | 204 | ? | ? | 156 | 174 | 118 | 128 | 112 | 114 |
| F03 | 228 | 231 | 162 | 176 | 198 | 204 | ? | ? | ? | ? | ? | ? | ? | ? |
| F04 | 235 | 238 | 161 | 175 | 201 | 204 | ? | ? | 174 | 182 | 118 | 124 | 111 | 113 |
| F05 | 231 | 236 | 161 | 162 | ? | ? | ? | ? | 169 | 174 | 118 | 124 | 111 | 112 |
| F06 | 235 | 238 | 176 | 178 | 202 | 203 | ? | ? | 171 | 174 | 118 | 124 | 110 | 111 |
| F07 | 229 | 231 | 160 | 162 | ? | ? | 248 | 250 | 167 | 179 | 118 | 124 | 111 | 113 |
| F08 | 232 | 238 | 174 | 176 | ? | ? | ? | ? | ? | ? | ? | ? | ? | ? |
| F09 | ? | ? | 160 | 162 | ? | ? | ? | ? | 180 | 185 | 118 | 124 | ? | ? |
| F10 | 235 | 238 | 157 | 165 | ? | ? | ? | ? | 156 | 156 | 118 | 124 | 111 | 113 |
| F11 | 231 | 238 | 160 | 162 | ? | ? | 246 | 248 | 171 | 178 | ? | ? | 108 | 112 |
| F12 | 229 | 229 | 162 | 176 | 201 | 204 | ? | ? | 172 | 182 | 118 | 124 | 112 | 117 |
| F13 | 238 | 240 | 173 | 175 | ? | ? | ? | ? | ? | ? | ? | ? | ? | ? |
| F14 | 231 | 240 | 162 | 174 | 206 | 207 | ? | ? | ? | ? | ? | ? | ? | ? |
| F15 | 229 | 235 | 164 | 174 | 198 | 204 | ? | ? | 157 | 165 | 118 | 124 | ? | ? |
| F16 | 232 | 238 | 165 | 178 | 198 | 204 | ? | ? | 174 | 177 | ? | ? | ? | ? |
| F17 | 231 | 236 | 165 | 178 | 201 | 204 | ? | ? | 180 | 185 | 118 | 124 | 114 | 115 |
| F18 | 233 | 237 | 163 | 176 | 201 | 204 | ? | ? | ? | ? | 119 | 125 | ? | ? |
| F19 | ? | ? | 166 | 166 | 198 | 204 | ? | ? | 157 | 166 | ? | ? | ? | ? |
| pop = Junaynah |  |  |  |  |  |  |  |  |  |  |  |  |  |  |
| J01 | 231 | 236 | ? | ? | ? | ? | ? | ? | 157 | 178 | 118 | 124 | 111 | 112 |
| J02 | 230 | 233 | 156 | 158 | ? | ? | ? | ? | 156 | 156 | ? | ? | ? | ? |
| J03 | 232 | 236 | 162 | 174 | 201 | 203 | 282 | 283 | ? | ? | ? | ? | ? | ? |
| J04 | 229 | 231 | 164 | 174 | 198 | 202 | 247 | 250 | ? | ? | ? | ? | ? | ? |
| J05 | 232 | 235 | 158 | 184 | 206 | 209 | 294 | 295 | 172 | 176 | 118 | 124 | ? | ? |
| J06 | 229 | 233 | 169 | 172 | 201 | 204 | 282 | 283 | 169 | 172 | 118 | 124 | 111 | 112 |
| J07 | 231 | 234 | 155 | 178 | ? | ? | 279 | 283 | ? | ? | ? | ? | ? | ? |
| J08 | 231 | 233 | 162 | 166 | ? | ? | ? | ? | 157 | 164 | 118 | 124 | ? | ? |
| J09 | ? | ? | 183 | 186 | ? | ? | 247 | 250 | 172 | 176 | 118 | 120 | 108 | 110 |
| J10 | ? | ? | 160 | 165 | 198 | 204 | 256 | 257 | 181 | 184 | 119 | 123 | ? | ? |
| J11 | 231 | 238 | 170 | 174 | 201 | 204 | 275 | 275 | 170 | 176 | ? | ? | ? | ? |
| J12 | 231 | 233 | 161 | 182 | 200 | 203 | 257 | 258 | ? | ? | ? | ? | ? | ? |
| J13 | 230 | 231 | 158 | 161 | 198 | 204 | ? | ? | 172 | 178 | 118 | 124 | ? | ? |
| J14 | 230 | 231 | 172 | 170 | 202 | 204 | ? | ? | 158 | 161 | 118 | 124 | 107 | 111 |
| J15 | 231 | 238 | 164 | 172 | 201 | 204 | ? | ? | 158 | 161 | 118 | 118 | ? | ? |
| J16 | ? | ? | 181 | 185 | 198 | 204 | 284 | 294 | 154 | 158 | 117 | 124 | ? | ? |
| J17 | ? | ? | 161 | 182 | 201 | 204 | ? | ? | 156 | 186 | 118 | 125 | 108 | 111 |
| J18 | 231 | 233 | 162 | 174 | 201 | 207 | ? | ? | 161 | 163 | 118 | 125 | 105 | 112 |
| J19 | 231 | 232 | 160 | 174 | 200 | 203 | 268 | 294 | 167 | 181 | 116 | 128 | 111 | 112 |
| J20 | ? | ? | 169 | 171 | ? | ? | 283 | 284 | 169 | 172 | ? | ? | 106 | 112 |
| J21 | 232 | 235 | 156 | 169 | ? | ? | 293 | 294 | 158 | 185 | 118 | 125 | 100 | 113 |

**Supplementary 2: a. Scoring diagrams**


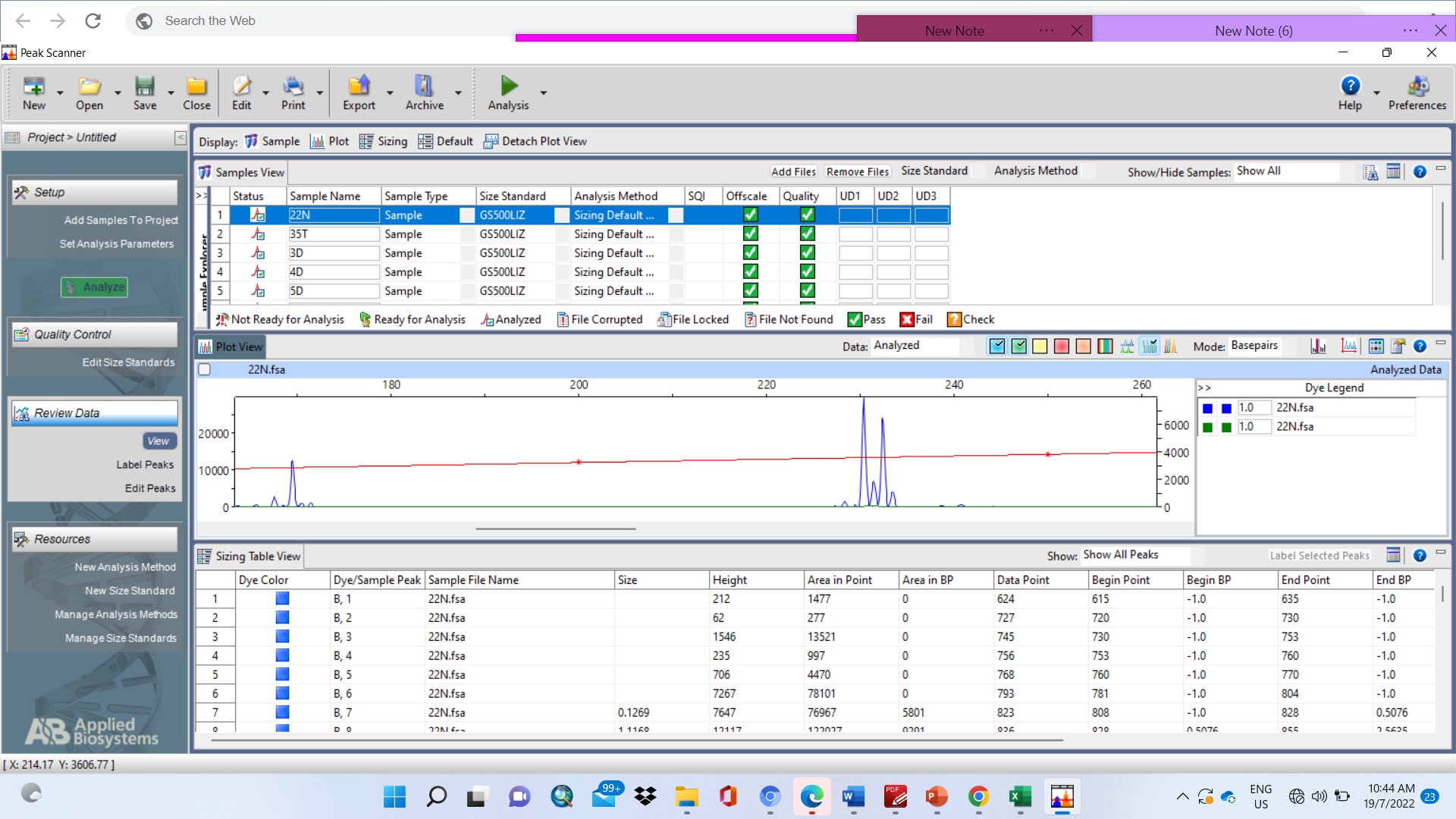


1. **Scoring diagram from peak scanner software**


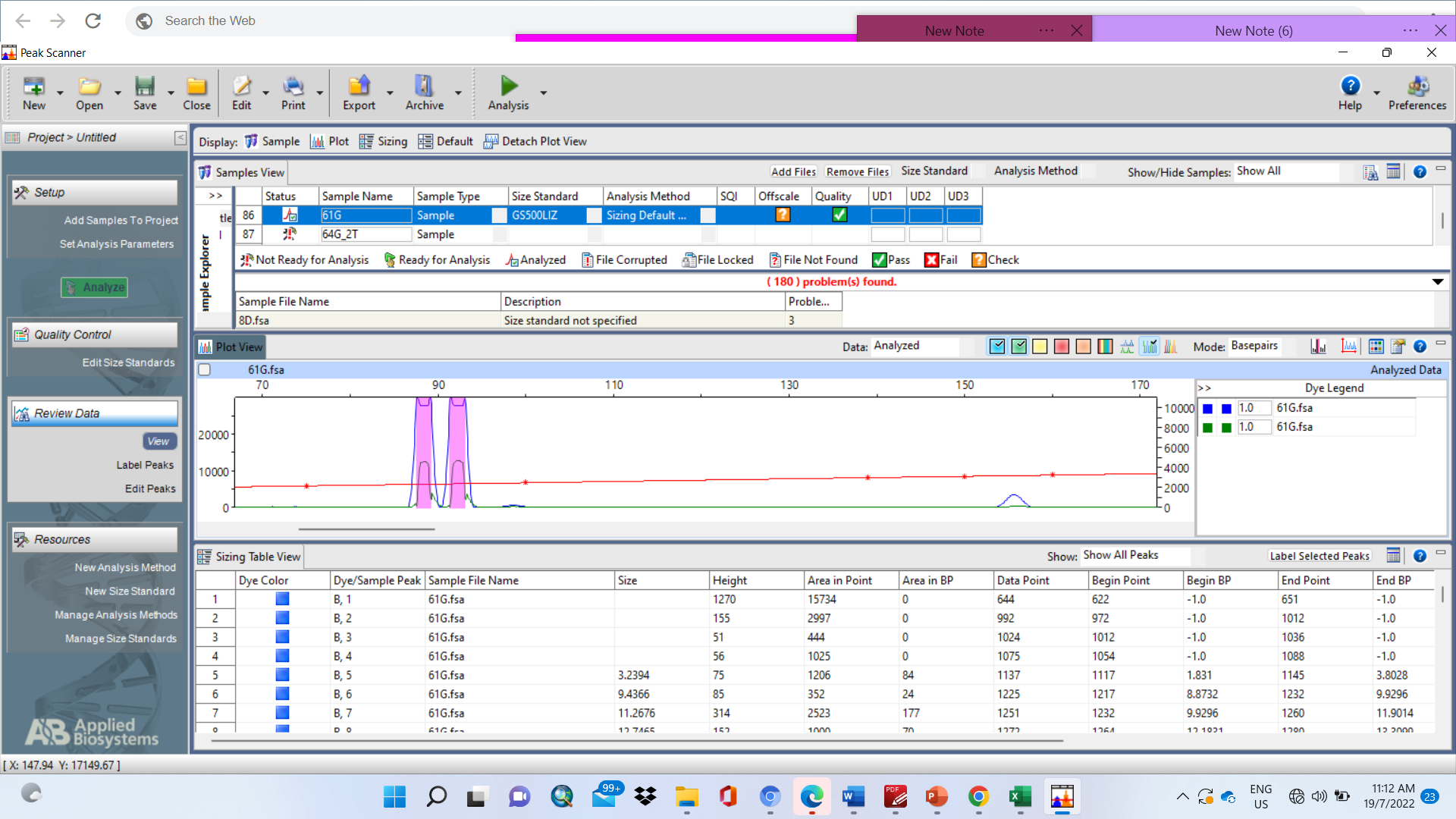


1. **Scoring errors example.**
